# Modeling individual self-protective behavior during epidemics

**DOI:** 10.1371/journal.pcbi.1014252

**Published:** 2026-05-08

**Authors:** Geonsik Yu, Michael Garee, Mario Ventresca, Yuehwern Yih

**Affiliations:** 1 Edwardson School of Industrial Engineering, Purdue University, ‌‌West Lafayette, Indiana, United States of America; 2 Air Force Institute of Technology, Wright-Patterson AFB, Dayton, ‌‌Ohio, United States of America; 3 Purdue Institute of Inflammation, Immunology, and Infectious Diseases, Purdue ‌‌University, West Lafayette, Indiana, United States of America; Animal and Plant Health Inspection Service, UNITED STATES OF AMERICA

## Abstract

Protecting public health from infectious diseases requires collective action, as individual behaviors—such as vaccination and mask-wearing—directly influence disease dynamics. During the COVID-19 pandemic, unexpected public responses often undermined the effectiveness of interventions, highlighting the need to understand collective behavioral patterns and motivations to design more effective mitigation strategies. This study presents an agent-based simulation model that captures how individuals adjust self-protective behaviors based on evolving opinions about disease risk and examines how these decisions interact with external factors, such as public health interventions, to shape collective outcomes. To improve the representativeness of the simulated population, multiple datasets were integrated to generate artificial populations. Model behavior was then calibrated against selected observed patterns, acknowledging that this calibration is partial and subject to model assumptions. Simulation experiments were conducted under varying conditions, including optional non-pharmaceutical interventions (NPIs), heterogeneous responses to pro- and anti-intervention messaging, and alternative vaccine eligibility policies. Results suggest that decision-making patterns may vary across demographic groups and that interactions between individual behaviors and external influences can substantially affect disease dynamics within the model. The simulation framework reproduces patterns consistent with observed phenomena, such as reduced mask-wearing following the lifting of mandates for vaccinated populations and higher infection burdens among economically disadvantaged populations. The framework suggests plausible mechanisms through which such patterns may emerge under the model assumptions. The study also suggests subpopulations likely to experience greater pressure under NPI mandates and highlights how vaccine eligibility strategies may enhance disease control when behavioral responses are considered. Overall, this work underscores the importance of integrating behavioral dynamics into epidemic response planning, while recognizing that findings are contingent on model structure and assumptions.

## Author summary

Effective control of infectious disease outbreaks, such as COVID-19, depends not only on medical interventions but also on how people choose to adopt protective behaviors like vaccination and mask-wearing. This study introduces an agent-based simulation model that captures how individuals’ opinions and decisions evolve in response to public health measures and information from media. By using real-world data to reflect diverse populations, the model explains why certain groups—e.g., economically disadvantaged communities—face higher infection risks. The findings offer insights for designing public health strategies that account for behavioral differences across populations. This work emphasizes the need to incorporate human behavior and decision-making into epidemic response planning to improve the effectiveness of future disease mitigation efforts.

## Introduction

In modern societies, public health interventions must operate within legal and ethical constraints that prevent strict control over individual behavior. As a result, voluntary public participation becomes essential for the success of strategies such as vaccination campaigns and mask mandates. Yet, individuals’ responses to these measures often diverge from expectations due to a complex interplay of risk perception, social influence, institutional trust, and structural barriers [1–3]. These challenges highlight the critical need not only to understand the drivers of behavioral variation and decision-making, but also to incorporate these factors into epidemic models to better inform the design of effective mitigation strategies.

To address this need, we develop an agent-based simulation model that incorporates individuals’ changing perceptions of disease risk and their decision-making regarding self-protective behaviors. This work extends existing epidemiological research by providing a novel framework to connect the decision-making process behind individual behaviors and their collective impact on public health interventions. The aim of this research is to answer the following research questions: How do collective patterns of individual decision-making, influenced by information sources and demographic traits, affect the outcomes of disease mitigation efforts during a pandemic? How do different intervention strategies, such as adjusting vaccine eligibility policies, interact with these behavioral patterns and influence disease outcomes?

Our simulation experiments build on a model calibrated using survey data, along with WHO and CDC datasets. The model captures how individuals’ self-protective behaviors evolve over time—driven by dynamic opinions, perceived risk, resource constraints, and age—and how these individual decisions aggregate to influence epidemic outcomes both overall and across subpopulations. We conduct a series of simulation experiments to explore how alternative intervention designs interact with heterogeneous behavioral dynamics.

In the first set of experiments, we examined the combined and sequential effects of NPI mandates and vaccine introduction on epidemic outcomes. While both countermeasures contribute significantly to disease mitigation, their effectiveness is highly dependent on behavioral compliance. Consequently, the impacts of these interventions vary across different subgroups, highlighting the importance of accounting for behavioral heterogeneity in public health planning.

In the second set of experiments, we explored how increased receptivity to anti-intervention messages—concentrated within specific demographic groups—affects disease spread. Although the magnitude of message bias was held constant, we varied which subgroups (based on age or resource availability) were affected. The results show that this uniform modification produces highly asymmetric effects depending on the group involved. Younger individuals and middle-resource groups, in particular, had a greater impact on transmission due to higher contact rates or limited capacity for protective action. These findings illustrate how targeted misinformation or localized distrust can disproportionately undermine public health efforts.

In the final set of experiments, we evaluated three alternative vaccine eligibility strategies—open eligibility, exposure-based prioritization, and resource-based prioritization—compared to the baseline age-based rollout. While individuals’ willingness to vaccinate remained unchanged, all alternative policies reduced overall infection levels. This improvement was driven by (1) earlier access for a greater share of willing individuals and (2) prioritization of groups more likely to transmit disease or face barriers to other protective behaviors. Notably, delaying vaccination for older adults did not worsen their outcomes due to improved population-wide suppression of disease transmission. These findings highlight the complex tradeoffs involved in eligibility design and suggest that optimizing for early coverage among high-transmission or socioeconomically constrained groups can enhance public health outcomes—though such strategies must be weighed carefully against ethical and clinical aspects.

Altogether, our findings underscore the importance of incorporating individual-level decision-making into epidemic models. By capturing how evolving opinions and sociodemographic contexts shape behavior, our framework offers valuable insights for designing more effective public health strategies. This approach enables policymakers to better understand behavioral heterogeneity across population subgroups, assess intervention tradeoffs, and enhance preparedness for future public health crises.

## Related literature

Human behavior is increasingly recognized as a critical factor in the study of infectious diseases [4–6]. The COVID-19 pandemic, one of the most consequential global health crises in modern history, has renewed attention to how individual behaviors and decisions can substantially influence disease dynamics. In response, a growing body of research has focused on characterizing behavioral tendencies across diverse subpopulations and integrating behavioral dimensions into epidemiological models to better reflect real-world dynamics.

Several studies have examined how behavioral tendencies vary across social, cultural, and demographic groups, particularly during the COVID-19 pandemic. Research has shown that key drivers of self-protective behaviors—such as risk perception [7–9], prosocial motivation [9,10], and trust in public health messaging [7,10–12]—can vary significantly across demographic groups. These differences have important implications, as public health interventions may elicit highly heterogeneous responses across different subpopulations. This underscores the need to better understand the behavioral responses of diverse demographic groups and the underlying drivers of those responses, so that strategies can be carefully designed and targeted—particularly in populations with varied sociodemographic backgrounds.

In response to the growing recognition of behavior’s role in epidemic dynamics, numerous modeling studies have integrated human behaviors and decision-making into infectious disease simulations [4,6,13]. Some approaches modify transmission rates based on behavioral adaptation [13–15], while others use agent-based models to capture individual responses to disease prevalence or public policies. For example, individuals may adjust social contact rates [16,17] or delay vaccination based on perceived risk [18]. These studies have demonstrated that incorporating such feedback can significantly alter epidemic outcomes and improve the realism of simulations. Their models highlight how behavioral diversity and adaptive responses complicate disease control and must be accounted for in effective public health planning.

Beyond capturing behavioral outcomes alone, a growing body of work has focused on the underlying processes that shape those behaviors—particularly opinion dynamics [19]. Notably, continuous shifts in public opinion [20,21] and increasing politicization [22] of relevant issues during the COVID-19 pandemic underscore the importance of incorporating opinion dynamics into infectious disease models. Early opinion dynamics models were typically developed in abstract or generic settings, emphasizing how beliefs evolve through peer influence to produce social conformity or polarization, without specific reference to real-world domains [23–26]. Recent research has adapted these frameworks to infectious disease contexts, where individual beliefs—shaped by social networks, institutional trust, and exposure to (mis)information—influence decisions such as vaccination or mask-wearing [5,15,19,27–29]. These models often co-evolve disease and opinion states, demonstrating how collective attitudes, beliefs, or opinions can either amplify or suppress transmission. By modeling the interplay between belief systems and disease dynamics, this line of work advances our understanding of collective behavior in public health crises.

While a growing number of models have incorporated behavioral aspects and public opinion into infectious disease dynamics—often acknowledging the heterogeneous nature of underlying populations—many studies rely on fixed behavioral assumptions [30,31], homogeneous or simplified characteristics of the underlying population [27,28], or lack real-world data integration when linking opinion, behavior, and disease dynamics [27–29,32]. Additionally, some frameworks link opinion trends directly to behavioral outcomes without explicitly modeling individual-level decision-making, thereby overlooking the complex processes by which individuals evaluate risk, respond to social influence, and act within structural constraints [15,27–30]. As a result, such models often fall short in capturing how individuals dynamically form and revise their decisions in response to evolving personal and social conditions. This gap limits their ability to reflect the nuanced pathways through which collective behaviors emerge and shift during real-world epidemics—particularly within subgroups that are poorly represented by population-level averages.

In contrast, our work demonstrates the value of explicitly modeling how individual decisions are shaped by evolving opinions and social contexts. By linking belief systems to behavior at the individual level, our approach enhances the realism of behavioral modeling and offers a framework for evaluating policy impacts in socially diverse populations. This provides a foundation for future research aimed at developing more adaptive, context-aware public health strategies.

To address these limitations, this study presents an agent-based simulation model that explicitly links individuals’ self-protective decisions to dynamically evolving personal opinions, as well as factors such as age and resource availability. By simulating how opinions are formed, influenced, and updated over time—and how they subsequently drive behavior during an outbreak—the model enables a detailed analysis of how heterogeneous decision-making patterns across demographic groups shape the effectiveness of public health interventions. This framework provides a valuable tool for evaluating mitigation strategies in socially diverse populations, offering insights especially relevant to real-world epidemics, where individual compliance is shaped by complex and evolving social contexts.

## Model and methods

We propose an agent-based simulation framework that integrates opinion dynamics and disease dynamics. The simulation framework is built upon the assumption that each agent makes decisions regarding self-protective behavior during epidemic events as informed by the agent’s opinion about the situation. The following subsections explain the components and procedures of the simulation model.

Note that this section highlights only selected key features; all remaining technical details are provided in S1 Appendix. The source code of the simulator and the relevant input data are available in the archived GitHub repository [33].

### Model overview

The simulation model considers a networked population of *N* individual agents. The population size (*N*), which defines the primary scale of the model, is set to 20,000, meaning that interactions among 20,000 individual agents are simulated and the resulting system-level dynamics are observed. This population size is strategically chosen to be sufficiently large to avoid premature disease extinction driven by small-population effects and stochastic variability, while ensuring computational feasibility given the resource constraints of this study.

**Fig 1:**
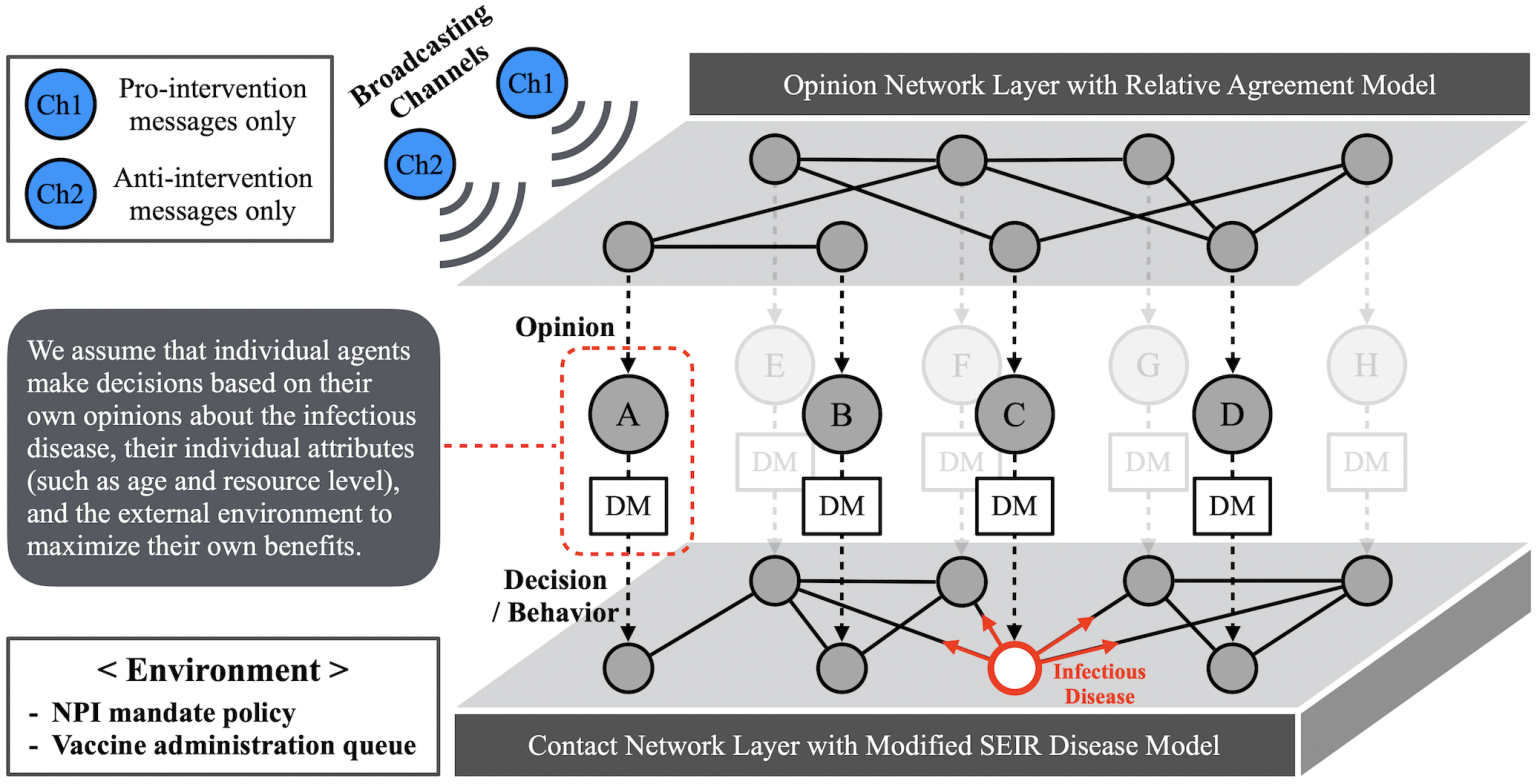
Schematic of the two-layer networked simulation model. Each individual agent (A, B,..., etc.) has attribute values that define its age, resource level, opinions, behavior, and status throughout a simulation run. Their opinions are updated through interactions in the opinion network layer via the relative agreement model. There are two broadcasting channels that transmit pro-intervention and anti-intervention messages, respectively. Agents make decisions regarding their NPI compliance and vaccination willingness based on the given situation and their opinions. This decision-making process is represented by the DM (Decision-Making) boxes in the figure. The infectious disease spreads throughout the contact network at the same time. Individual decisions regarding self-protection affect the spread of the disease.

The model consists of two network layers: an opinion network and a contact network (Fig 1). On the opinion network layer, agents update their opinions about infectious diseases through interactions with neighboring agents and through exposure to mass media messages. Opinion updates follow a relative agreement framework [15,26]. We assume that all agents are potentially exposed to two mass media broadcasting channels: one transmitting pro-intervention messages and the other transmitting messages not advocating interventions. These channels function as opinion leaders or authorities that exert disproportionate influence on public opinion [24]. The opinion network is static and fixed over time, representing stable social ties through which individuals repeatedly exchange beliefs and information.

An individual’s opinion, however, does not solely determine their self-protective behavior. At each time step, agents make decisions regarding non-pharmaceutical intervention (NPI) compliance and vaccination willingness based on a combination of personal attributes (e.g., age and resource availability, reflecting economic affluence), prevailing policies, system-level conditions, and their current opinions. These decisions, in turn, influence disease transmission on the contact network layer.

On the contact network layer (bottom layer of Fig 1), agents may transmit the disease to one another through close interpersonal contact. Disease progression follows a modified SEIRS framework [34]. The contact network is regenerated at each time step while preserving the same degree configuration, reflecting dynamic mixing patterns that are directly relevant for disease transmission. Given that the COVID-19 pandemic represents the most recent large-scale outbreak for which behavioral and epidemiological patterns are well documented, the baseline testbed for this study is designed to reproduce selected features of the pandemic observed in the United States. Accordingly, the disease modeled in this study shares key characteristics with SARS-CoV-2. Model parameters are calibrated using data from the early stages of the COVID-19 pandemic (2020–2021) to enhance the realism of the baseline simulations, which serve as the foundation for subsequent experiments.

At the system level, the simulation environment evolves over time and shapes individual agents’ decisions and behaviors. Environmental factors include NPI mandate policies, vaccine availability and eligibility policies, and system-level constraints such as vaccination capacity. Vaccine eligibility expands over time following an age-prioritized framework in the baseline scenario, consistent with U.S. COVID-19 vaccination policies. Vaccination is additionally constrained by limited administration capacity, which restricts the rate at which willing and eligible agents can be vaccinated.

**Fig 2:**
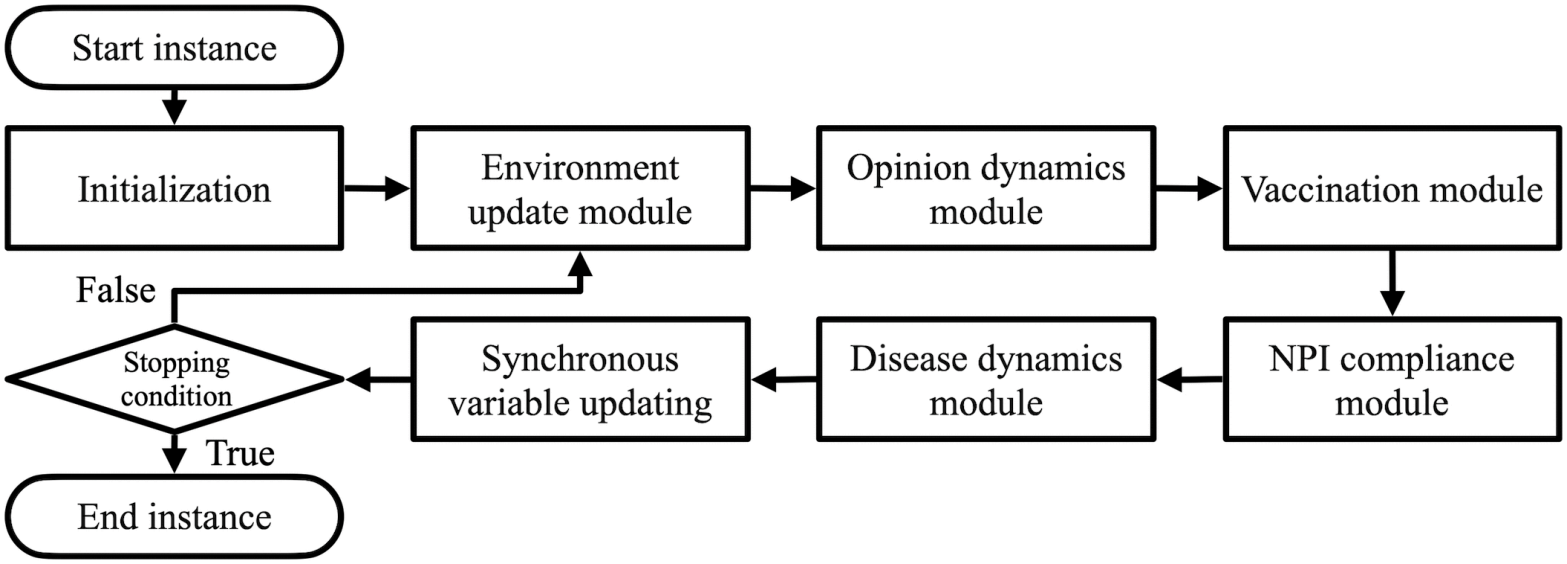
Simulation procedure flow for a single instance.

Each simulation instance proceeds iteratively following initialization. Time is discretized in daily steps. At each time step, the following modules are executed in sequence: (1) environment, (2) opinion dynamics, (3) vaccination, (4) NPI compliance, and (5) disease dynamics. The overall simulation flow is illustrated in Fig 2. Simulation outputs are recorded after completion of all iterations.

All agent attributes governing opinion dynamics, disease dynamics, and decision-making are listed in Tables A and B in S1 Appendix. Full details regarding attribute initialization, update rules, and submodel implementations are also provided in the same appendix, following the ODD (Overview, Design concepts, Details) protocol for agent-based models [35]. In the following section, we describe key details of the individual submodels corresponding to the modules shown in Fig 2.

We note that the model adopts a one-directional structure in which disease dynamics do not feed back into opinion formation or behavioral decision-making. Instead, opinions influence individual decisions, which in turn affect disease transmission. This simplifying assumption allows us to isolate the role of opinion-driven decision-making and reduces computational burden by enabling a two-stage calibration procedure, in which opinion-related parameters are fitted first and disease-related parameters are fitted subsequently. This divide-and-conquer strategy reduces the dimensionality of the parameter space at each stage and improves the tractability of fitting the model to observed trends.

On the other hand, this assumption excludes potential feedback from epidemic conditions (e.g., local infection prevalence in an agent’s neighborhood) to media channels or individual risk perception, which may influence model outcomes. This limitation is discussed further in the Discussion section.

## Submodels

### Environment module.

The environment module governs time-varying factors that globally affect all agents and shape their decision-making. In our simulation framework, this module manages three key aspects: policies regarding NPIs, the vaccine rollout schedule, and eligibility criteria. These factors are exogenous to individual interactions but influence behavioral incentives and constraints throughout the simulation.

First, public health policies regarding NPIs evolve over time. Initially, NPIs are presented as voluntary guidelines until time *t*_1_, when these guidelines become mandatory. When the mandate is imposed, noncompliance incurs a penalty for agents. After time *t*_2_, when vaccines become available, mandates are partially relaxed such that penalties apply only to unvaccinated agents. During the mandate period, a penalty value (*C*^(*p*)^) is introduced as a perceived cost of noncompliance and is incorporated into agents’ decision-making processes. The binary variable $X_{k,t}^{\left(p\right)}$ indicates whether this penalty is considered in agent *k*’s decision-making at time step *t* or not. The following equation defines how this binary variable is de*t*ermined within the environment update module.


$$\begin{array}{c} \begin{array}{c} X_{k,t}^{\left(p\right)}=\left\{ \begin{array}{cc} 0, & \begin{array}{c} \text{if }t<t_{1}\;\text{(NPI mandate not applied yet)}\\ \text{or }(t\geq t_{2}\text{ and }V_{k,t}=1)\;\text{(Mandate lifted, agent }k\text{ is vaccinated)} \end{array}\\ 1, & \begin{array}{c} \text{if }t_{1}\leq t<t_{2}\;\text{(NPI mandate in effect)}\\ \text{or }(t\geq t_{2}\text{ and }V_{k,t}=0)\;\text{(Mandate lifted, agent }k\text{ is not vaccinated)} \end{array} \end{array}\right. \end{array} \end{array}$$ (1)

Vaccine availability and eligibility criteria constitute another layer of the simulation environment. The vaccine administration capacity (𝒦) represents the maximum number of vaccines that can be administered per day. This capacity is zero prior to vaccine introduction (*t*_3_), increases to ${𝒦}_{1}$ at the *t*ime of introduction, and subsequently increases to ${𝒦}_{2}$ as vaccine availability expands (*t*_4_). This constraint limits the rate at which willing and eligible agents can receive vaccination. Vaccine eligibility is governed by a rolling eligibility framework tha*t* expands over time. In the baseline scenario, eligibility follows an age-based priority system, consistent with the backbone of U.S. COVID-19 vaccination policies in most U.S. states. Detailed availability and eligibility schedules used in our simulation are provided in Section 5.1 of S1 Appendix. The corresponding U.S. vaccine rollout data are presented in S4 Appendix.

#### Opinion dynamics module.

This module updates agents’ opinions and their corresponding uncertainty values on two different topics using update rules adapted from the relative agreement (RA) model [26]. As the RA model describes, each agent has an opinion value and the corresponding uncertainty value. The uncertainty describes how uncertain or stubborn an agent is about its own opinion. Low uncertainty indicates that an agent is more stubborn in its own opinion and less likely to change it. In our simulation framework, each agent *k* holds two opinion values: the perceived probability of infection when not vaccinated and not complying with NPI guidance (${\tilde{P}}_{k,t}^{\left(0,0\right)}$), and the perceived probability of experiencing side effects when vaccinated (${\tilde{P}}_{k,t}^{\left(s\right)}$). $U_{k,t}^{\left(0,0\right)}$ and $U_{k,t}^{\left(s\right)}$ are the corresponding uncertainty values. These two perceived probabilities are subsequently used by the decision-making module when agents decide on vaccination willingness and NPI compliance.

Each individual agent first randomly selects a source of information to update their opinion. The two attributes, $M_{k}$ and $F_{k}$ determine which channels or individual agents will be selected. With probability $\left(M_{k}\cdot F_{k}\right)$, the source of information is the pro-intervention media channel, while with probability $\left(M_{k}\cdot (1-F_{k})\right)$, it is the anti-intervention media channel. With probability $\left(1-M_{k}\right)$, agent *k* chooses to learn from its neighbors in the opinion network instead of the media channels. Since our opinion network is unweighted and undirected, the agent selects a neighbor uniformly at random from its set of neighbors. In the following opinion-updating equations, *P*_ext_ and *U*_ext_ denote the opinion and the corresponding uncertainty value of the selected entity.

The attributes $M_{k}$ and $F_{k}$ are fixed over time for each agent and are independently drawn at initialization from Beta distributions defined on [0,1]. These distributions capture heterogeneity in agents’ media engagement tendencies and relative preference for pro-intervention versus anti-intervention content. The shape parameters of these Beta distributions are treated as model calibration parameters, allowing the aggregate influence of media consumption and information bias to be adjusted to result output patterns similar to observed patterns in the data.

Equations 2 define the opinion updating rules. Equations 2 are used for opinion updates prior to *t*_3_, while the same equations with the superscript (*s*) replacing (0,0) is applied starting from *t*_3_, reflecting the change in the environment.


$$\begin{array}{c} \begin{array}{c} {\tilde{P}}_{k,t+1}^{\left(0,0\right)}={\tilde{P}}_{k,t}^{\left(0,0\right)}+\mu_{1}^{\left(0,0\right)}(h/U_{\text{ext}}^{\left(0,0\right)}-1)(P_{\text{ext}}^{\left(0,0\right)}-{\tilde{P}}_{k,t}^{\left(0,0\right)}) \end{array} \end{array}$$ (2a)

$$\begin{array}{c} \begin{array}{c} U_{k,t+1}^{\left(0,0\right)}=U_{k,t}^{\left(0,0\right)}+\mu_{2}^{\left(0,0\right)}(h/U_{\text{ext}}^{\left(0,0\right)}-1)(U_{\text{ext}}^{\left(0,0\right)}-U_{k,t}^{\left(0,0\right)}) \end{array} \end{array}$$ (2b)

$$\begin{array}{c} \begin{array}{c} \text{where }h=\min(U_{k,t}^{\left(0,0\right)},U_{\text{ext}}^{\left(0,0\right)}+|{\tilde{P}}_{k,t}^{\left(0,0\right)}-P_{\text{ext}}^{\left(0,0\right)}|) \end{array} \end{array}$$ (2c)

$$\begin{array}{c} \begin{array}{c} \;-\max(-U_{k,t}^{\left(0,0\right)},|{\tilde{P}}_{k,t}^{\left(0,0\right)}-P_{\text{ext}}^{\left(0,0\right)}|-U_{\text{ext}}^{\left(0,0\right)}) \end{array} \end{array}$$ (2d)

*h* represents the overlap between the opinion range segments of agent *k* and the selected external source, where the segments are defined by placing their opinion values at the center and determining their width using the corresponding uncertainty values. The μ parameters are learning rates that govern the magnitude of opinion and uncertainty updates during interactions.

Under repeated interactions, the RA model tends to produce stabilization of opinion values over time, as agents gradually converge toward locally consistent beliefs within their social and media environments. In our framework, this implies that opinion dynamics do not change substantially after reaching a stable state. Parameters governing opinion updates primarily influence the early stages of the simulation.

#### Vaccination and NPI compliance modules.

This module determines, at each time step, whether individual agents choose to comply with non-pharmaceutical interventions (NPIs) and whether they are willing to receive vaccination, given their current beliefs, attributes, and policy environment. Agent *k*’s NPI compliance ($C_{k,t}$) and vaccination willingness ($W_{k,t}$) are encoded as Boolean variables, taking the value *True* if the agent complies with NPIs or is willing to be vaccinated, and *False* otherwise. If a willing agent is selected from the vaccination queue, the agent’s disease status ($D_{k,t+1}$) is updated accordingly.

To represent individual agents’ decision-making regarding vaccination and NPI compliance, we adopt a structured decision framework in the form of a sequential decision tree [36,37]. The sequential decision tree is composed of decision nodes and chance nodes. At decision nodes (squares), agents compare the expected values of available actions and select the branch with the highest value. At chance nodes (circles), uncertainty is represented by computing a weighted average of downstream branch values according to their associated probabilities. The computation proceeds backward from the leaf nodes to the root, ensuring that decisions at each node are evaluated based on the expected values of their subsequent outcomes. The final output of the decision tree is the decision at the root node that is corresponding to the branch with the largest assigned value.

At each time step, agents evaluate available self-protective actions by sequentially considering perceived risks, personal attributes, and prevailing policies. This framework is particularly suited to modeling situations in which individuals face multiple, distinct self-protection options—in our case, combinations of vaccination decisions and NPI compliance—and must weigh their respective costs and benefits. The sequential structure provides an interpretable way to encode how agents translate beliefs and contextual constraints into behavioral choices. The design of the decision trees used in this module is further inspired by theories of insurance and utility, as self-protection measures against infectious diseases resemble insurance decisions in that individuals may incur immediate costs to reduce potential future losses [37].

**Fig 3:**
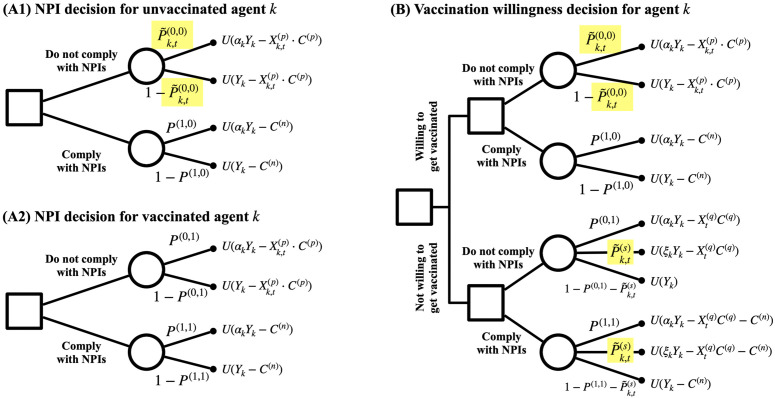
Decision trees for individual agents. Individual agents make decisions through three decision trees. An agent’s compliance with NPIs, such as wearing a mask or maintaining social distancing, is determined through decision trees (A1) and (A2), depending on its vaccination status. If an agent is eligible for vaccination, it decides on its willingness to get vaccinated using the decision tree **(B)**. Only two probability values (highlighted) are based on an agent’s perception that changes over time: ${\tilde{P}}_{k,t}^{\left(0,0\right)}$ and ${\tilde{P}}_{k,t}^{\left(s\right)}$, while other probability values are fixed at a certain value for simplification. This is to minimize the complexity of the model and have only one dynamic opinion value closely related to each of the two self-protection behaviors: NPI compliance and vaccination willingness.

Agents make decisions at each time step using the three decision trees illustrated in Fig 3. Decision trees (A1) and (A2) govern agents’ NPI compliance choices, conditional on their vaccination status, while decision tree (B) determines vaccination willingness for vaccine-eligible agents. Vaccination-related decisions are evaluated starting at time *t*_3_ when vaccine administration begins. Prior to vaccine introduction (*t* < *t*_3_) *t*he vaccination decision module is skipped. The decision process for vaccination willingness jointly incorporates vaccination and NPI compliance choices, reflecting their role as interdependent self-protection strategies whose perceived benefits and costs are evaluated together.

Decisions within the trees are based on agents’ perceived risks and decision-relevant costs. In particular, agents use perceived probabilities of infection and vaccine side effects to evaluate expected outcomes. Among the probability terms appearing in the decision trees, only two values represent dynamic agent perceptions that evolve over time: the perceived risk of infection in the absence of any self-protection measures (${\tilde{P}}_{k,t}^{\left(0,0\right)}$) and the perceived probability of experiencing severe vaccine side effects (${\tilde{P}}_{k,t}^{\left(s\right)}$). All other probability terms are held fixed for model parsimony. Under this design, each self-protection behavior is closely linked to a single evolving opinion variable that governs its outcome. The perceived probabilities used in the decision tree represent agents’ subjective beliefs rather than objective epidemiological risks and therefore need not coincide with the true characteristics of the disease.

Decision outcomes also depend on costs associated with policy compliance and system constraints. Agents who comply with NPIs incur a compliance cost (*C*^(*n*)^), while agents who do not comply may incur a non-compliance penalty (*C*^(*p*)^) depending on the prevailing policy environment. Additionally, agents may experience disutility from congestion in the vaccine administration queue (*C*^(*q*)^), which can reduce vaccination willingness when demand exceeds capacity. To capture heterogeneity in vaccination motivation, agents who are willing to tolerate queue congestion are prioritized by the vaccination queue. If capacity remains after serving the eligible and prioritized agents, additional willing agents are selected at random. This mechanism introduces differentiation in realized vaccination timing without explicitly modeling strategic queue behavior.

Branches of the decision tree associated with infection or severe vaccine side effects reduce agents’ resource availability ($Y_{k}$) through age-dependent modifiers based on the agent’s age ($A_{k}$). We assume that infection-related modifiers ($\alpha_{k}$) reduce an agent’s resource availability mildly for agents younger than 65 ($\alpha_{k}=\alpha_{k}^{<65}$) and more substantially for agents aged 65 and older ($\alpha_{k}=\alpha_{k}^{65+}$). These reductions reflect differential economic or health impacts across subpopulations and influence subsequent decision-making. Similarly, severe vaccine side effects ($\xi_{k}$) are modeled as reductions in resource availability, with age-dependent effects that differentially impact agents’ resource levels.


$$\begin{array}{c} \begin{array}{c} \alpha_{k}=\left\{ \begin{array}{cc} \alpha_{k}^{<65}, & \text{if }A_{k}<65,\\ \alpha_{k}^{65+}, & \text{if }A_{k}\geq 65, \end{array}\right. \end{array} \end{array}$$ (3)

Agents are assumed to make decisions based solely on their vaccination status and do not observe latent disease states such as exposure or natural immunity. Vaccinated agents are assigned a recovered disease status, and agents who are currently infectious are assumed to recognize symptoms and therefore do not seek vaccination. The decision-making model is also intentionally simplified. Agents maximize expected utility using a linear utility function, and intertemporal discounting is not considered. While this abstraction omits many real-world cognitive and behavioral complexities, it enables transparent interpretation of how relative perceptions of risk and cost shape vaccination and NPI compliance decisions. Limitations of this approach are discussed later in the article.

#### Disease dynamics module.

This module governs individual-level disease state transitions in the contact network layer. At each time step *t*, each agent *k* updates its disease state $D_{k,t}$ according to the transition rules summarized in Table 1. State transitions are evaluated at the agent level based on the agent’s current disease state, its local contact environment, and vaccination.

**Table 1:** State transitions and expected binomial transition sizes for internal disease dynamics of standalone midge habitat agents.

From ($D_{i,t}$)	To ($D_{i,t+1}$)	Condition	Probability
$D_{i,t}=S$	$D_{i,t+1}=E$	${𝒩}_{i,t}^{I}\neq \emptyset$	$1-\prod_{j\in {𝒩}_{i,t}^{I}}(1-\lambda_{ijt})$
$D_{i,t}=E$	$D_{i,t+1}=I$	–	σ
$D_{i,t}=I$	$D_{i,t+1}=R$	–	γ
$D_{i,t}=R$	$D_{i,t+1}=S$	–	ω
$D_{i,t}\in \{S,E,I,R\}$	$D_{i,t+1}=R$	Vaccination at *t*	–

If agent *k* is in the susceptible state ($D_{k,t}=S$), its risk of exposure depends on its infectious neighbors in the contact network at time *t*. Let (${𝒩}_{k,t}^{I}$) denote the se*t* of infectious neighbors of agent *k* at time *t*. When this set is nonempty, the agent transitions from suscep*t*ible to exposed (S→E) through independent Bernoulli trials with probability ($\lambda_{kjt}$) for each infectious neighbor *j*. That is, a susceptible agent transitions to the exposed state if at least one such trial succeeds. This procedure is equivalent to sampling a single aggregate transition probability as shown in the table.

Agents in the exposed state ($D_{k,t}=E$) progress to the infectious state (E→I) with probability σ. Infectious agents ($D_{k,t}=I$) recover (I→R) with probability γ, while recovered agents ($D_{k,t}=R$) lose immunity and return to the susceptible state (R→S) with probability ω. Vaccination acts as an exogenous event that immediately transitions agents in any disease state to the recovered state, reflecting vaccine-induced immunity.

Note that we assume compliance with NPI guidance reduces the transmission rate if at least one of the two interacting agents follows the guidance [15,38]. Specifically, if either of the two individuals in contact wears a face mask, the reduced transmission rate is applied to that interaction. Conversely, if neither complies with the guidance, the original transmission rate, $\lambda_{0}$, is used. The following equation describes how this rule is implemented in our simulator:


$$\begin{array}{c} \begin{array}{c} \lambda_{kjt}=\left\{ \begin{array}{cc} (1-\rho )\lambda_{0}, & \text{if }C_{i,t}=True\text{ or }C_{j,t}=True\\ \lambda_{0} & \text{otherwise.} \end{array}\right. \end{array} \end{array}$$ (4)

where ρ represents the reduction rate provided by self-protection measures.

Our model also incorporates external changes in disease spread to account for the first peak in new cases. Multiple studies have discussed various external factors around the first peak of the COVID-19 pandemic, including changes in intervention policies (e.g., restriction change) and shifts in people’s behavior (e.g., vacation, holiday seasons, and large political gatherings and rallies before the U.S. presidential election) [3,39–41]. To incorporate such temporary changes in a simplified manner, our model allows for adjustments in the intervention’s effectiveness (ρ) during a specified period ($\left[t_{a},t_{b}\right]$) by a factor of (1−δ), representing reduced effectiveness.

### Data and model fitting

The primary purpose of the simulation model is to establish testbeds that replicate key patterns observed during the early COVID-19 pandemic in the United States. These testbeds are then used for simulation-based analyses under varied assumptions to examine interactions among opinion dynamics, individual-level decision-making under heterogeneous personal circumstances, and disease spread. Model calibration is therefore employed to identify plausible parameterizations that reproduce essential empirical patterns, rather than to construct a predictive or digital-twin model.

The model fitting described in this section was conducted under the following seven setups: *C*^(*p*)^ = 100, 150, 200, 250, 300, 350, and 400. The trends in NPI compliance levels and vaccination are primarily driven by relationships among cost-related parameters, as agents make decisions to minimize potential losses due to disease. Fixing one cost parameter therefore systematically determines the others once model calibration is complete. To construct testbeds across systematically distinct parameter sets and avoid highly similar configurations, we fix *C*^(*p*)^—the penalty cost for non-compliance with the NPI mandate—at these selected values and proceed with calibration. S2 Appendix illustrates the seven calibrated models with different *C*^(*p*)^ values used in the process, showing that they are generally similar with only minor differences. All results presented in the main article are based on averages across these seven calibrated models.

**Fig 4:**
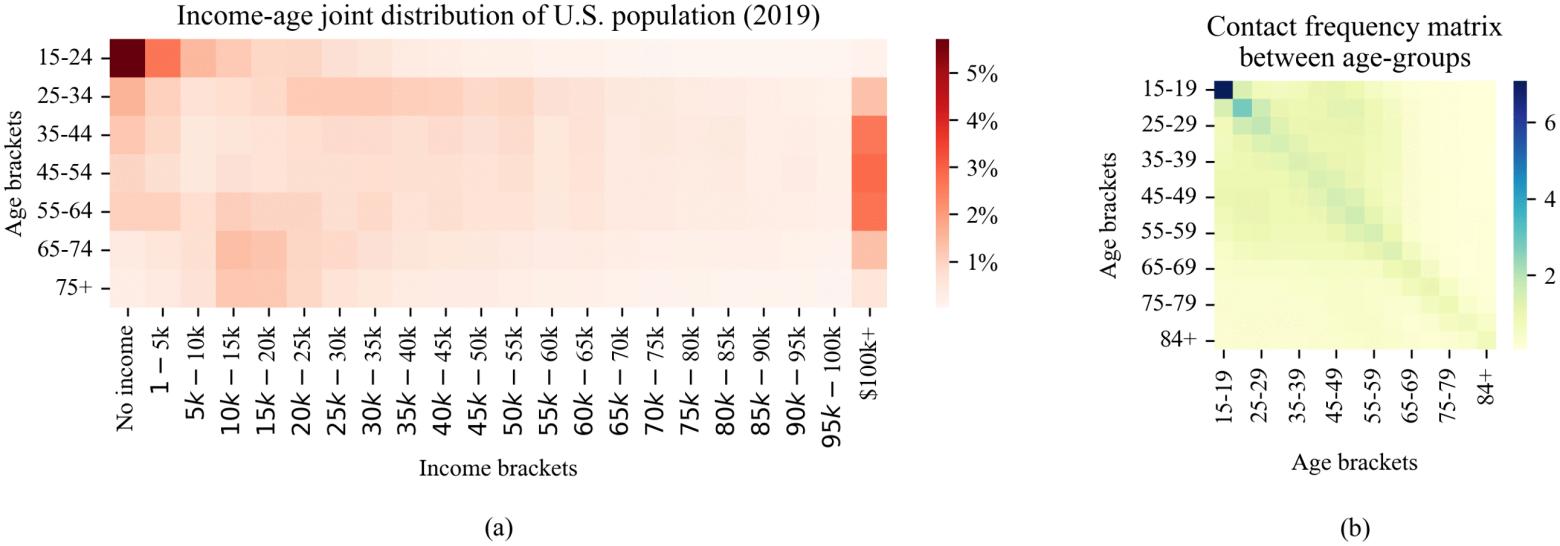
Two datasets incorporated in the model for generating the population characteristics. Plot (a) visualizes the U.S. Census Bureau dataset about the joint distribution of income and age, and plot (b) presents a heatmap of contact frequency matrix between age groups.

Table 2 presents the data sources used to generate an artificial population that reflects selected social traits of the U.S. population during the pandemic, as well as the datasets employed for model calibration. Fig 4 visualizes the two datasets used to generate the artificial population [42,43]. The first heatmap shows the joint income–age distribution of the U.S. population, and the second heatmap illustrates contact frequencies between age groups. Note that the income distribution data are based on self-reported survey responses and therefore reflect individuals’ subjective perceptions.

**Table 2:** Dataset descriptions and their sources.

Usage	Data Description	Data Source
Model Calibration	Responses from the YouGov survey to the question, ‘How often have you worn a face mask outside your home (e.g., when using public transport, visiting a supermarket, or going to a main road)?’ as a proxy for people’s opinions on preventive interventions.	YouGov [21]
	New cases of COVID-19	WHO [44]
	Number of people vaccinated at least one time per 100 people	CDC [45]
Population Generation	Income-age joint distribution of the US population (2019)	US Census Bureau [42]
	Contact matrix between age-groups	[43]
Others	Initial vaccination administration date in the USA and the date as of which all U.S. states had opened vaccine eligibility to residents aged 16 and over.	[46–49]
	Reporting rate of COVID-19 infection in USA	[50]

We calibrate the model to three empirical time series derived from the data sources listed in Table 2: (1) average NPI compliance, (2) weekly new confirmed cases, and (3) the cumulative number of individuals who have received at least one vaccine dose. For scoring, simulated daily outputs are aggregated into non-overlapping 7-day periods, and the resulting 7-day averages are compared against the corresponding 7-day averaged empirical observations over the same time frames. Model fitting is performed by minimizing the root mean square error (RMSE) between empirical observations and simulated sequences at corresponding time steps. A basin-hopping optimization framework is employed, in which Latin hypercube sampling (LHS) with the minimax correlation criterion is used to generate multiple starting points for global search, followed by sequential quadratic programming (SQP) for local optimization [15,51,52]. For each parameter tuple, 30 stochastic simulation replications are conducted to compute the RMSE.

As mentioned earlier, the fitting process is conducted in two stages to reduce the dimensionality of the parameter space and the associated computational burden. In the first stage, parameters governing NPI compliance levels and vaccination trends are calibrated against their respective datasets. For each of the seven *C*^(*p*)^ setups, 5,000 parameter tuples are generated using LHS and evaluated, after which the top 100 performing tuples are selected for local optimization. In the second stage, the remaining epidemiological parameters are calibrated to align the average model output with observed disease dynamics, while parameters identified in the first stage are held fixed. This stage follows the same procedure, starting with 5,000 LHS-generated tuples and refining the top 100 through local search.

This calibration strategy is enabled by the assumed one-directional structure of the model, in which disease dynamics do not feed back into individuals’ decisions regarding NPI compliance or vaccination. Additional details on model fitting and extended results are provided in S2 Appendix. Calibration parameters and fitted values are presented in S3 Appendix. Sobol sensitivity analysis results for the fitted parameter values are provided in S5 Appendix.

## Results

In this section, we first present the models calibrated to U.S. data collected during the COVID-19 pandemic. Then, the outcomes of various alternative scenarios derived from the baseline testbed model are presented, where each is designed to explore different aspects of policy interventions, how individual-level decision-making interacts with these interventions, and the tendencies observed in different demographic groups.

For the alternative scenarios, we first examine cases where key interventions—NPI mandates and vaccines—are removed either individually or in combination, to assess their relative impact on individuals’ decision-making and disease spread. Next, we investigate the influence of heterogeneous responses toward pro- and anti-intervention messages within specific subgroups, examining how increased receptivity to anti-intervention messaging alters compliance and disease dynamics. This also helps determine whether distrust of NPIs among certain subgroups can undermine disease mitigation efforts more than similar inclinations in other groups. Finally, the impact of different vaccine eligibility management rules is examined with alternative vaccine eligibility scenarios.

Across all scenarios, we track changes in NPI compliance, vaccination rates, and overall disease spread, both at the population level and within specific demographic subgroups. We tally the total number of compliance behavior in two categories: compliance among the unvaccinated and compliance among the vaccinated. This is because vaccination is the other self-protective decision against disease that an individual can make.

### Baseline scenario

**Fig 5:**
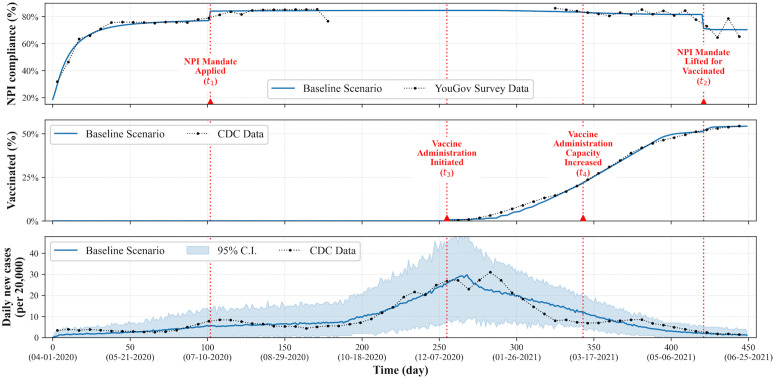
Simulation results of the calibrated baseline model: average NPI compliance level trend over time (top), trend (%) of the population vaccinated (middle), and daily new case trend (bottom). The simulation results based on the calibrated parameter tuples are presented (210 instances in total for visualization). The four vertical dotted lines mark significant events of the baseline scenario.

Fig 5 visualizes the simulation results of models calibrated to U.S. data collected during the COVID-19 pandemic. As shown in the plots, the simulation spans from day 0 (April 1st, 2020) to day 450 (June 25th, 2021), covering the early period of the COVID-19 pandemic. Our agent-based simulation model mimics the trends in behavior among the U.S. population and the trend of disease spread, and we use the models as our baseline scenario for further experiments with varied scenarios.

Four key policy and epidemiological milestones are marked by vertical dashed lines. At *t*_1_, an NPI mandate is introduced, leading to a rapid increase and subsequent stabilization of compliance levels. When the NPI mandate is applied, a penalty for non-compliance is incorporated into each agent’s decision-making process. At *t*_3_, vaccine administration is initiated, followed by a gradual increase in vaccination coverage that accelerates after *t*_4_, corresponding to an expansion in vaccine administration capacity. Finally, at *t*_2_, NPI mandates are lifted for vaccinated individuals.

Note that many details are obscured due to the simplifications in our modeling approach. For example, the introduction of the Delta variant to the U.S. population in mid-March 2021 is not included in our model, and the so-called ‘Delta variant peak’ falls outside the simulation time span of our study [53]. This is another limitation of our model, as variants of COVID-19 are known to have distinct characteristics, such as increased transmission rate per contact. There are also state-wise differences in changes to NPI policies and vaccination schedules, which are similarly obscured, as the model is fitted to nationwide aggregate data.

### Impact of NPI mandates and vaccine administration

This section aims to assess the impact of two public health interventions—NPI mandate and vaccine introduction—on disease spread through comparing baseline scenario and the three variant scenarios: no NPI mandate, no vaccine, and neither NPI mandate nor vaccine introduction as shown in Table 3. In the No-NPI-mandate scenarios, NPIs are provided as guidelines only and are not enforced through mandates. Public opinion regarding NPIs continues to evolve through interpersonal interactions and media influence, as in the baseline scenario. However, no penalty for non-compliance is applied in agents’ decision-making processes, and compliance is driven solely by perceived infection risk and compliance-related costs. In the no-vaccine scenario, the NPI mandate is imposed over the same period as in the baseline scenario, including the penalty for non-compliance, but no vaccine is introduced. Agents’ protective behavior therefore relies entirely on NPI compliance decisions. In the last scenario with neither NPI mandate nor vaccine introduction, agents are not subject to any enforced public health interventions. NPIs are available only as voluntary guidelines, and no penalties for non-compliance are imposed. As in the baseline scenario, public opinion continues to evolve through interpersonal interactions and media influence.

**Table 3:** Alternative scenario table.

Scenarios	Descriptions
Baseline	(1) NPI mandate is imposed for the time period from *t*_1_ to *t*_2_. (2) Vaccine administration rolls out from *t*_3_ and expands its capacity from *t*_4_. Rolling eligibility is used based on age.
No NPI Mandate	(1) NPI is provided as a guideline only and is not imposed as a mandate. (2) Vaccine administration rolls out as in the baseline scenario.
No Vaccine	(1) NPI mandate is imposed for the time period as in the baseline scenario (from *t*_1_ to *t*_2_.) (2) No vaccine introduction.
No NPI & No Vaccine	Absence of both interventions. (1) NPI is given as a guideline only and is not imposed as a mandate. (2) No vaccine introduction.

We assess the impacts of the two interventions (NPI mandates and vaccination) by comparing patterns across the four scenarios. The effect of NPI mandates is evaluated by comparing the baseline scenario with the no-NPI-mandate scenario, as well as the no-vaccine scenario with the no-intervention scenario. Similarly, the effect of vaccine introduction is evaluated by comparing the baseline scenario with the no-vaccine scenario, as well as the no-NPI-mandate scenario with the no-intervention scenario. By comparing the patterns observed across scenarios, we provide insight into the formation of collective decision-making patterns under the simulation setup and assumptions.

**Fig 6:**
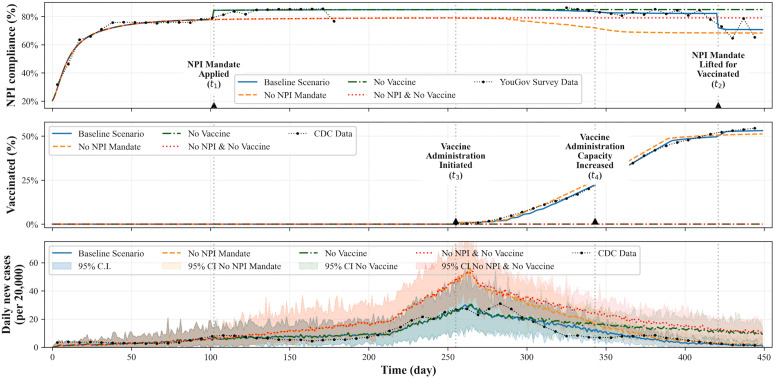
Simulation results of the calibrated baseline model and three alternative scenarios: (1) no NPI mandate, (2) no vaccine, and (3) no NPI mandate and no vaccine at the same time. Since the random seeds are strictly managed over simulation runs, we can observe the clear bifurcating branches between scenarios around day 100 and day 250 in the first and third rows.

Fig 6 presents the simulation results of the calibrated baseline models and three alternative scenarios. As in the previous section, weighted average trends from 30 simulation instances across 7 different calibrated models are used to visualize the overall results for all the scenarios. In the alternative scenarios, relevant parameters are adjusted to simulate each assumed situation. Random seeds are strictly controlled across simulation runs to isolate the impact of changes in different scenarios from randomness. Clear bifurcating branches between scenarios can be observed around day 100 and day 250 in the first and third rows. From the results, we can see that the combination of the two interventions is effective in disease mitigation.

From the first row of Fig 6, average NPI compliance decreases when vaccines are available. This pattern emerges from the model’s decision-making framework, in which agents may substitute vaccination for NPI adherence based on perceived costs and benefits. Although the model allows individuals to adopt both strategies simultaneously, the simulated population more frequently favors one over the other under the simulation setup. In contrast, when vaccines are not introduced, compliance remains higher, as NPIs represent the primary available protective measure. The last row of the figure shows that both interventions—NPI mandates and vaccination—substantially influence disease dynamics within the model, with mandates reducing peak infection and vaccines contributing to longer-term control, while agents follow the same decision-making logic across scenarios.

We note that additional simulation results without media channels or opinion leaders are provided in S6 Appendix. In this scenario, no entities actively intervene to influence public opinion or shape consensus regarding the disease, NPIs, or vaccine side effects. While this scenario represents a stricter interpretation of “no intervention,” it is included in S6 Appendix because it serves primarily as additional exploratory scenario rather than a core scenario aligned with our main research questions. Information dissemination through media channels and influential actors is pervasive in modern social settings and difficult to eliminate entirely.

**Fig 7:**
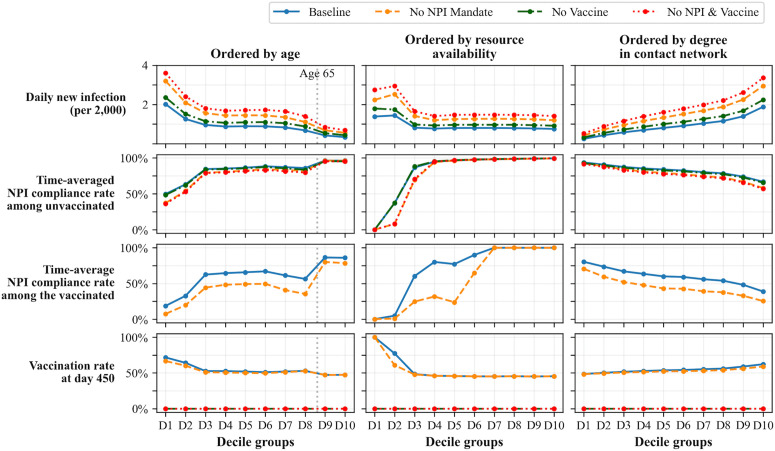
Simulation results (1) with different scenarios across each decile group. From the plots, we can observe that both the NPI mandate and vaccine introduction significantly reduced disease spread by comparing the daily new cases across decile groups in different scenarios (1st row, lower is better). The plots in the 2nd, 3rd, and 4th rows (higher is better) show how agents make decisions regarding self-protection against infectious diseases based on their characteristics or conditions.

Fig 7 presents the simulation results for different scenarios across decile groups (D1, D2, ..., D10), categorized based on three criteria: age, resource availability, and degree in the contact network. Each decile represents 10% of the simulated population, corresponding to 2,000 agents per group, with D1 denoting the lowest decile and D10 the highest for the respective attribute. Accordingly, D1 represents the youngest group in the age-based categorization (column 1), the lowest resource-availability group in the resource-based categorization (column 2), and the least connected group in the contact network-based categorization (column 3). Similarly, D10 represents the oldest, highest resource-availability, and most connected groups in their respective categorizations. Decile stratification is used to capture systematic heterogeneity across the ‌‌population while maintaining interpretability, allowing us to compare outcomes between subgroups.

In each row of Fig 7, the following metrics are presented for each decile group. The first row shows the average number of daily new infections per 2,000 agents within each decile group. The second row shows the time-averaged NPI compliance rate among unvaccinated agents, defined as the total number of unvaccinated and NPI-compliant agents summed over time divided by the total number of unvaccinated agents summed over time. The third row shows the time-averaged NPI compliance rate among vaccinated agents, defined as the total number of vaccinated and NPI-compliant agents summed over time divided by the total number of vaccinated agents summed over time. Finally, the fourth row shows the vaccination rate achieved at the end of the simulation.

The first-row subplots of Fig 7 show that both the NPI mandate and the introduction of vaccination substantially reduced disease spread across all population subgroups, with relatively greater impacts observed in specific demographic groups. In particular, the subplots indicate that both interventions lead to larger reductions in the number of new infections among individuals who are younger, have lower resource availability, and have a higher degree of contact in the network, relative to the baseline scenario. These groups exhibit the lowest numbers of new cases globally under the intervention scenarios. Although the interventions also effectively reduce new infections among older individuals, such as those aged 65 and older, the absolute reduction in the number of new cases is relatively smaller in this group.

The second- and third-row subplots illustrate how individuals’ NPI compliance tendencies are influenced by their characteristics and by policy conditions. The first-column subplots shows that compliance behavior is relatively higher among the older group (D9 and D10), whose health-related risk is potentially greater, and relatively lower among the younger group. This is due to our assumption that older individuals perceive a higher potential loss from contracting an infectious disease and because resource availability is generally lower among the younger generation. The gap between the older and younger groups grows larger among those who are vaccinated (row 3) compared to the gap among the unvaccinated (row 2). This suggests that older individuals are more likely to keep NPI compliance after getting vaccination in our configuration. The gap between the older group and the rest group grows greater if there is no NPI mandate. Among individuals under 65, there is heterogeneity in NPI compliance like in the D1, D2, D7 and D8 groups. This tendency is driven by the average resource availability within each decile group by age. For example, the resource availability level is significantly smaller for the youngest 20% group.

In the second column, we observe even more pronounced patterns. This result is expected, as a cost–benefit perspective lies at the core of agents’ decision-making in our model. In our framework, becoming infected leads to a proportional reduction in an agent’s resource availability. Consequently, agents with greater resource availability experience larger absolute losses from infection. When an agent’s resource availability is sufficiently high, compliance with NPIs (and vaccination) is always beneficial, regardless of the magnitude of the non-compliance penalty. In contrast, when resource availability is very low, the penalty imposed by NPI mandates is insufficient to induce behavioral change. As a result, individuals in the lowest resource-availability group are relatively insensitive to variations in mandate-related penalties. For agents in the intermediate resource-availability group, however, the non-compliance penalty plays a pivotal role. In this range, the penalty is large enough to alter the relative costs and benefits of compliance, effectively resulting changes in agents’ decision-making. Consequently, NPI mandates have the largest marginal impact on compliance behavior in this group.

The fourth and final row shows the vaccination rate at the end of the simulation runs. With respect to age, the D9 and D10 groups exhibit slightly lower vaccination uptake than the remaining age groups. This pattern arises because older individuals place greater weight on the potential impact of vaccine side effects ($\xi_{k}$), given their age-related vulnerability, although the overall difference relative to other groups is modest. Patterns driven by resource availability, which is similar to those observed for NPI compliance, also re-emerge for vaccination behavior. Agents with lower resource availability display distinct vaccination responses, reflecting differences in how self-protection strategies are evaluated. Because vaccination in the model involves only the perceived risk of side effects and does not impose additional monetary or compliance costs, it represents a more economically favorable self-protection option for individuals in the D1 and D2 resource-availability groups. Consequently, these groups tend to exhibit relatively higher vaccination uptake despite their lower overall resource levels.

Note that vaccination tendencies across groups differ from those reported for the U.S. population during the COVID-19 pandemic. This discrepancy arises mainly because model calibration was performed using aggregate behavioral tendencies rather than subgroup-specific temporal sequences. We revisit and discuss this limitation in the Discussion section.

### Heterogeneous responses to intervention-related messages

In this section, we explore alternative scenarios in which certain subgroups of people are more inclined to respond to anti-intervention messages than to pro-intervention messages. These scenarios provide insights into how different subpopulations may engage differently with public health guidance and the pandemic itself when there is a same degree of inclination toward the anti-intervention messaging in each group.

Such variations in response patterns were observed during the COVID-19 pandemic [11,54,55]. Psychological tendencies such as institutional skepticism, divergent risk perceptions, and reactive devaluation (where individuals perceive authorities or channels promoting pro-intervention messages as untrustworthy or biased) can shape how individuals engage with public health guidance [56]. While these tendencies differ in their underlying mechanisms, existing studies highlight that variations in responses to public health messaging were more pronounced among specific demographic groups rather than being uniformly distributed across the population during the COVID-19 pandemic [11]. These insights underscore the importance of exploring the alternative scenarios proposed in this section.

In our baseline setup, the parameters defining individual agents’ information-collecting behaviors, such as channel usage, are distributed homogeneously across the population. In this section, this homogeneity is modified by assigning a specific demographic group of agents a relative distrust of pro-intervention messages, making them more likely to learn from anti-intervention messages instead. Eight different settings are examined, each involving a random selection of half of the individuals within a specific quartile (Q1, Q2, Q3, or Q4), defined by either age or resource availability. The selected individuals are then influenced to adopt behaviors aligned with anti-intervention messaging. The remaining population outside of these selected agents keeps their media consumption attributes the same as in the baseline models. By experimenting with these varied scenarios, we examine whether such heterogeneity in the responses to messages can be more impactful and potentially more harmful in terms of disease mitigation among certain demographic groups.

To implement biased behavior in the selected population, their learning rates for anti-intervention messages is increased by 100% while decreasing their learning rates for pro-intervention messages by 90%. Additionally, their uncertainty regarding anti-intervention messages is reduced by 50%. These changes make them learn anti-intervention beliefs more quickly and more intensely. Note that the degree of these changes is arbitrarily selected, as we lack empirical evidence to precisely match the parameters of the relative agreement model to real-world phenomena. However, the direction of these changes aligns with our intended assumptions, making the direction of change in these alternative scenarios meaningful, rather than the size of their impact.

For the results, three different measures are tracked to assess the changes in each trend: (1) the percentage change in the average compliance level before day 100, (2) the percentage change in the vaccinated population on day 450, and (3) the percentage change in the average number of new cases. These metrics are presented as bar charts to facilitate comparison among different scenarios in their respective plots.

**Fig 8:**
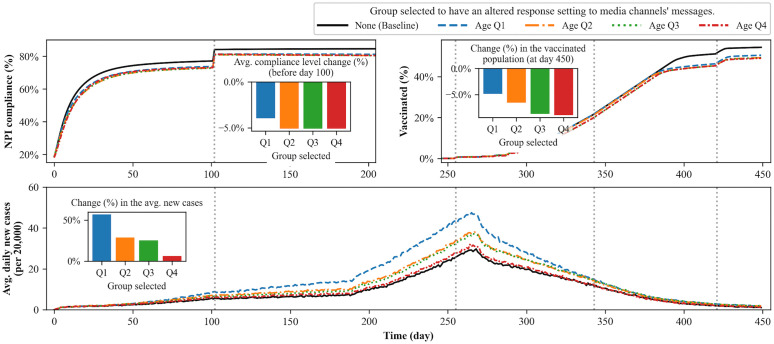
Simulation results for scenarios in which a specific age demographic shows an inclination toward anti-intervention messages. The solid curve shows the simulation results of the baseline scenario, in which none of the agents are selected to have an altered response setting to media channels’ messages. Three inserted bar charts show the percentage changes in measures caused by different setups compared to the baseline model. The later part (beyond Day 200) of the NPI compliance plot and the early part (before Day 250) of the vaccination plot are omitted, as they remain the same level in those periods. The plots show that the inclination toward the anti-intervention messages among the younger generation (Q1) causes the greatest change in disease spread.

Fig 8 presents the results of scenarios in which groups of agents, selected based on age, exhibit altered inclinations toward intervention-related messages. The results indicate that the presence of a distrusting group among the younger age cohort (1st quartile) leads to the largest increase in average new infections (56.86%), whereas such an inclination toward the anti-intervention messages among older cohorts (3rd and 4th quartiles) results in a much smaller impact on disease spread (25.5% and 6.26% increases, respectively). One reason for the greater effect of changed inclination and reduced acceptance of pro-intervention messages among younger generations is the relationship between contact degree and age. The underlying contact network data reveal that younger individuals have more extensive contact exposure compared to older generations, and their interactions are predominantly within the same age groups. That is, if distrust toward pro-intervention messages exists among the younger generation, they are more likely to encounter others with the same distrust, as their within-group contacts are frequent. Conversely, if such distrust is present among the older generation, they are less likely to meet others sharing the same belief because their interactions are more likely to span different generations.

**Fig 9:**
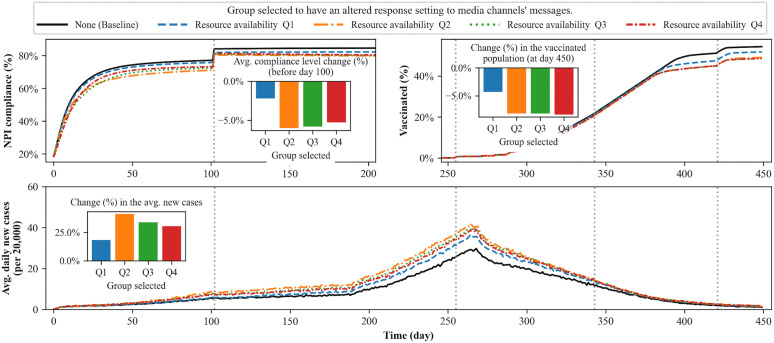
Simulation results for scenarios in which the inclination toward anti-intervention messages is concentrated within specific quartiles of the population by resource availability. The inserted bar charts and omitted sections follow the same structure as Fig 8. The plots show that the inclination toward the anti-intervention messages among the middle-resource groups (Q2 and Q3) causes significant changes in NPI compliance behavior and leads to a greater impact on disease spread.

Fig 9 shows that the inclination toward the anti-intervention messages among the middle-resource group has the greatest impact on disease spread: a 41.1% increase when such inclination is present in the 2nd quartile and a 33.9% increase when it is in the 3rd quartile. While changes in NPI compliance and vaccination in Fig 8 appear relatively homogeneous, individual agents’ behavioral patterns show distinct differences among the alternative scenarios in Fig 9. This is primarily because alternative scenarios in Fig 9 are directly connected to resource availability which has greater impact in agents’ decision-making process.

The changed inclination among the 2nd and 3rd quartiles leads to a greater decrease in NPI compliance compared to the other two quartiles, which in turn contributes to a greater spread of disease. The decline in vaccination is more significant when the distrust against the intervention exists among the 2nd, 3rd, and 4th quartiles. However, the trend in average daily new cases converges similarly across all scenarios, indicating that the impact of the decline in vaccination rates after day 370 is limited.

Further results detailing the changes caused by a changed inclination toward the messages among specific demographic groups, analyzed in decile groups, are provided in S2 Appendix as these results are relatively marginal.

### Vaccine eligibility management

In the baseline scenario, it is assumed that an age-based rolling eligibility (or sequential age-based prioritization) policy is used for vaccine administration. The policy is a widely studied and applied public health strategy for prioritized vaccine distribution as it distributes the direct protection to those who are with a higher risk of severe health outcome from the infectious disease [57–60].

This section explores alternative vaccine eligibility management policies and examines how individual-level decision-making in our model interacts with these changes. We test the following three alternative eligibility rules: (1) open eligibility, (2) exposure-based rolling eligibility, and (3) resource-based rolling eligibility. For open eligibility, vaccine access is granted to all individuals unconditionally, provided they are willing to receive the vaccine. For exposure-based rolling eligibility, vaccine eligibility is determined by an individual’s risk of exposure, represented by their degree in the contact network. Those with higher connectivity are prioritized for vaccination earlier. Similar to age-based rolling eligibility, access gradually expands over time until everyone becomes eligible. Lastly, for resource-based rolling eligibility, vaccine access is prioritized for individuals with vulnerable socioeconomic status. In our simulation, those with low resource availability are granted early eligibility for vaccination. As in the previous scenario, eligibility expands gradually over time, with the highest resource availability group receiving access in the final stage.

**Fig 10:**
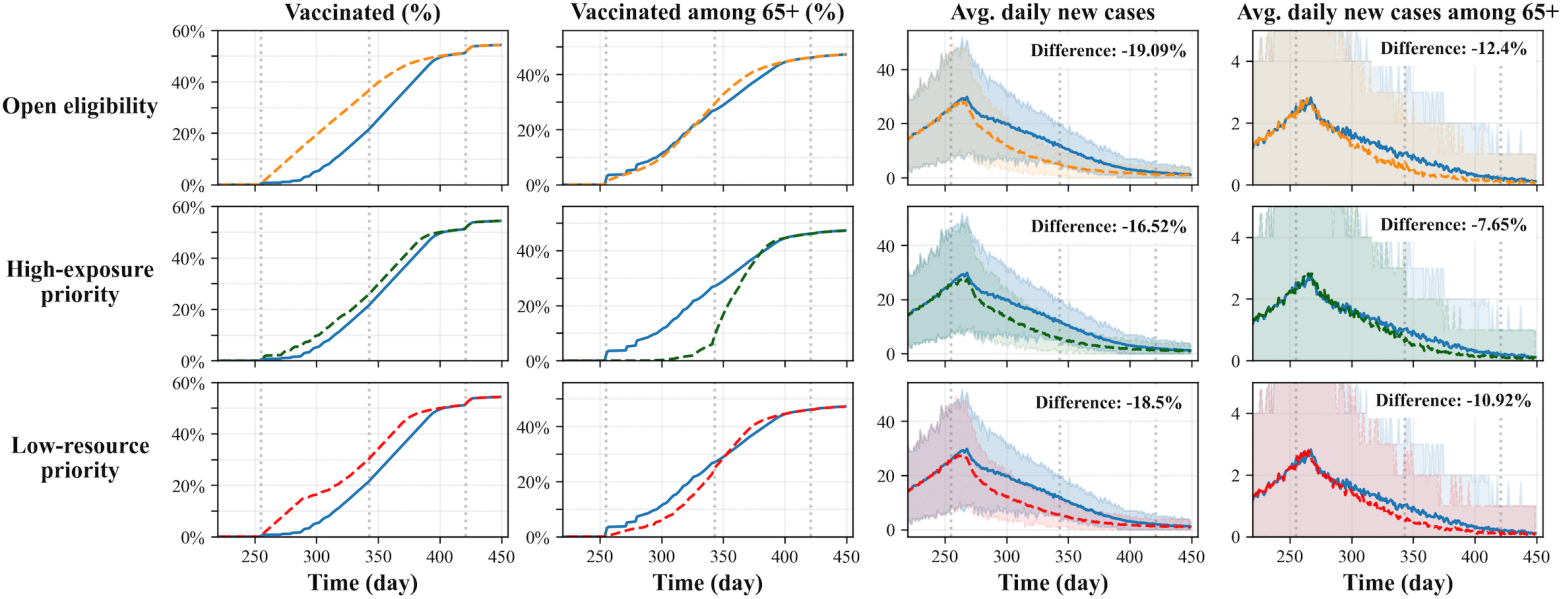
Simulation results comparing the outcomes of the baseline scenario (blue curve) with those of alternative vaccination policies: open eligibility (top), high-exposure priority (middle), and low-resource priority (bottom). The results show that, under our simulation settings, alternative scenarios outperform age-based vaccine prioritization by enabling higher vaccine administration during the early stage. This leads to a smaller peak in new cases. Although the alternative policies delay vaccination for populations at higher risk of severe outcomes, they more effectively reduce overall disease spread by mitigating transmission among high-exposure populations and those with limited resources, who may have lower motivation for self-protection. Notably, the impact is limited to the faster distribution of vaccines to the willing population and does not change the total number of individuals willing to be vaccinated. This leads to convergence in vaccination trends around *t* = 400.

Fig 10 presents results for the baseline scenario alongside various alternative vaccine eligibility management strategies. The figure illustrates changes in vaccination trends in the first and second columns and the corresponding impact on disease spread in the third and fourth columns. The results indicate that the baseline model’s age-based rolling eligibility policy is outperformed by all alternative policies in terms of total new cases over time.

There are two key reasons for this change in disease spread. First, there is an increased number of willing and eligible individuals in the early vaccination stage. In all three alternative vaccination policies, a larger proportion of eligible individuals are also willing to be vaccinated, leading to a higher level of community protection. Second, there is a shift in the characteristics of the early vaccinated population. While the baseline model prioritizes older individuals, who are also more likely to engage in self-protective behaviors against the disease, the exposure-based rolling eligibility prioritizes individuals who contribute more significantly to disease transmission. Similarly, the resource-based rolling eligibility prioritizes individuals for whom NPI compliance is less affordable. These differences in the composition of the early vaccinated population also influence the overall spread of the disease.

Note that the vaccination rate among the 65 + age group slows down significantly under exposure-based rolling eligibility but not under the other two policies. Additionally, in all three alternative scenarios, the average number of new cases among the 65+ age group decreases compared to the baseline model. This suggests that overall disease mitigation can be improved by prioritizing the vaccination of populations who are not the most likely to develop severe symptoms or by delaying vaccination for older individuals while still effectively protecting them. However, we emphasize the need for extreme caution when interpreting this result for two reasons. First, the outcome is highly dependent on our model assumptions. It is possible that older individuals are not as self-protective as our model assumes. Second, this improvement in overall disease mitigation comes at the cost of prolonged risk exposure for older individuals, who are more likely to experience severe health outcomes from infection.

## Conclusion

We developed an agent-based simulation model that incorporates people’s changing perceptions of the spreading disease over time and their individual decision-making. Our modeling approach enhances the understanding of how individuals’ choices interact with disease spread and public health interventions. Simulation experiments are conducted with multiple scenarios to better understand changes in various outcome patterns.

In the first experiment set, we assessed the impact of public health interventions by removing them from the baseline scenario. Simulation results show that both NPI mandate and vaccination are effective in disease mitigation, with the combination of both being particularly impactful. Both interventions reduce disease spread more effectively in younger, less wealthy, and more connected groups. Compliance behavior is generally higher among older individuals, but the reduction in new cases by interventions is more significant in younger groups. Vaccination patterns are shown to align with resource availability, with individuals in lower-resource groups more likely to opt for vaccination. Pressured compliance is more common among younger individuals and those with lower resource availability.

The second experiment set tested the impact of the inclination toward the anti-intervention messages within specific demographic groups, which is particularly relevant given the observed distrust during the COVID-19 pandemic. We modified the baseline setup by introducing the inclination toward the anti-intervention messages in a subset of agents. The results indicate that such heterogeneous response to media messages has a more significant impact on disease spread when distrust is present in younger individuals, those with middle-range resources, and those with higher contact rates.

The final set examined and compared three alternative vaccine eligibility policies against the baseline age-based rolling eligibility. Results show that all alternative policies outperform the baseline in reducing disease spread. This is due to a higher proportion of willing and eligible individuals being vaccinated early and changes in the characteristics of the early vaccinated population. However, caution is needed as the older population’s vaccination rate slows under alternative policies, and the improvement in disease mitigation comes at the cost of prolonged risk exposure for older individuals.

## Discussion

Our simulation experiments with the agent-based model fitted to the real-world data reveal several notable behavioral patterns among demographic subpopulations and suggest explanations for these patterns. First, our model provides a potential mechanism for why disease spread is more persistent among low-income groups [61–63] and among younger generations with higher connectivity [64,65], by modeling their motivations and decision-making. We also identify which demographic groups are more likely to change their compliance behavior by NPI mandates, and be potentially under the stress of such policy.

The NPI compliance patterns produced by our model are broadly consistent with empirical observations from the COVID-19 pandemic, with lower compliance observed among younger individuals and those with economic disadvantages, and higher compliance observed among older individuals and those with higher incomes [66–68]. In contrast, the vaccination patterns generated by the model differ from empirical observations. Multiple studies have reported that older and economically advantaged individuals were more likely to receive COVID-19 vaccination, whereas in our model, younger and economically disadvantaged groups exhibit higher vaccination uptake due to the assumed cost-free nature of vaccination [69–71]. Existing studies suggest that this divergence may be driven in part by lower confidence in vaccines and heightened concerns about potential side effects, such as myocarditis, among younger populations [72,73].

This divergence indicates that real-world COVID-19 vaccination uptake was shaped by drivers—such as differences in education levels, non-financial access barriers, historical and structural distrust, and cultural pressures—that are only partially represented or abstracted in our framework, rather than by cost–benefit considerations. Also, our model does not include drivers that would cause younger generations to fear or to overvalue vaccine side effects more than older generations. By design, our model emphasizes cost–benefit logic under simplified assumptions, which helps explain why alignment with empirical data is stronger for NPI compliance than for vaccination outcomes.

Another key contribution of this study is our simulation results on distrust in public health interventions across demographic groups. In the United States, distrust in public health measures manifested in various ways during the COVID-19 pandemic. The Tuskegee Syphilis Study has had a profound and lasting impact on African Americans’ trust in government-led health interventions, a pattern that seemingly persisted throughout the COVID-19 pandemic [74,75]. Political affiliation has also been shown to influence levels of distrust in public health measures during pandemic events [76,77]. Our findings suggest that while similar patterns of distrust can emerge across different subpopulations, their impact varies depending on demographic characteristics and decision-making tendencies. In some groups, the consequences of distrust may be significantly more severe, even when skepticism toward public health interventions is consistent across different scenarios. Once again, the collective patterns of individual decision-making drive these varied impacts of distrust.

Our simulation experiments on alternative vaccine eligibility policies also provide insights into why age-based rolling eligibility may be suboptimal from certain perspectives. The idea of providing indirect protection to vulnerable populations, such as older adults, is well-documented, particularly in the context of COVID-19 [57,78–80]. In fact, vaccinating healthcare workers and essential workers was a common practice in many U.S. states during COVID-19, even though the primary eligibility policy was typically age-based [80,81]. Existing studies primarily focus on the higher connectivity and transmission potential among younger populations to justify prioritizing their vaccination. Our simulation results suggest that, beyond connectivity, variations in motivation to comply with NPIs and adopt self-protective behaviors within different subgroups also contribute to the suboptimality of age-based rolling eligibility. However, it is important to interpret this result with caution, as improved overall disease mitigation could come at the cost of prolonged risk exposure for older individuals.

Our modeling approach, and consequently our results, have several limitations. The constraints outlined in the following paragraphs indicate that while our model is well-suited for capturing broad behavioral trends and deriving useful insights, it is not designed for precise predictive accuracy.

Some components of our model are based on assumptions and simplifications. Although our simulation population is constructed using real-world data and calibrated against observed pandemic outcomes, the model should not be considered a digital twin of society. Our model relies on the relative agreement framework for opinion dynamics, incorporating only two opinion-related parameters that evolve over time. Additionally, specific assumptions about media consumption behaviors are embedded in the model. The decision trees we employ assume that individuals make choices based solely on a selected set of factors, whereas, in reality, decision-making may be influenced by a broader range of social, political, and cultural considerations. The disease model we use is also a simplified SEIRS framework, which does not differentiate between varying disease severity levels or account for mortality.

We note that our model does not incorporate a feedback loop from disease dynamics to opinion dynamics, resulting in a unidirectional influence from opinions to disease outcomes. This simplification was made to balance model complexity, computational feasibility, and data availability. Such feedback can take multiple forms in real-world settings. For example, media messaging may adapt dynamically in response to changes in the number of infectious individuals, thereby influencing public opinion. Similarly, agents may update their risk perceptions based on direct observations of disease symptoms within their local social networks. While our simplification allows for clearer interpretation of how opinion dynamics influence decision-making and reduces computational burden, it limits the model’s ability to capture adaptive behavioral responses observed in real-world settings. Incorporating such feedback mechanisms could alter both the timing and magnitude of behavioral responses, particularly in alternative scenarios and in the heterogeneity observed across subpopulations, even if aggregate trends remain similar. Implementing this feedback while maintaining computational tractability represents an important direction for future work. In particular, insights from media studies and social psychology—such as how media narratives evolve during epidemics and how individuals respond to the experiences of close contacts—would be valuable for informing such extensions.

In the present study, our simplification may still reproduce compliance patterns observed in the U.S. population because compliance levels increase rapidly and stabilize early (around *t* = 50 in Fig 6), remaining relatively high until policy changes at *t* = 420. The relatively short simulation horizon further limits the role of dynamic feedback by maintaining a sustained level of caution in the population. However, empirical evidence from other countries suggests that compliance and disease dynamics can evolve differently [15]. In settings where behavioral consensus is not established early relative to epidemic peaks, feedback between disease dynamics and opinion formation may play a critical role in shaping outcomes. In such cases, incorporating feedback mechanisms would be essential for developing more realistic and informative simulations.

Our simulated artificial population and assumed physical connections also have certain limitations. For example, to reduce model complexity and computational burden, we do not explicitly incorporate family structure. As a result, the model may overestimate the financial constraints faced by younger individuals, as it does not account for the possibility that they may have access to their parents’ economic resources. Further heterogeneity can also be introduced into agents’ media consumption patterns and their varied responses to interventions to enhance the realism of our model. Additionally, the degree distribution of the contact network is held constant over time to mimic the structure observed in the underlying dataset; however, in real-world settings, contact patterns and effective connectivity may evolve dynamically in response to factors such as perceived infection risk, behavioral fear, and changes in public health policies. The potential impact of correlations between opinion connections and physical connections is another interesting aspect to explore when the above details of the underlying population are added. Further biological traits of virus, such as age-specific susceptibility, can also be explored. Addressing these limitations represents an important direction for future work.

Overall, our work demonstrates the value of modeling individual behaviors and opinions in understanding disease dynamics and policy impacts. While the model includes several simplifications, it offers useful insights into how people respond differently to interventions and how such diverse responses can influence the overall effectiveness of mitigation policies. Future work can build on this framework to improve realism and better support public health planning.

## Supporting information

S1 Appendix Detailed model description following the ODD protocol. This appendix provides a comprehensive description of our agent-based model following the ODD (Overview, Design concepts, Details) protocol. It details the entities, attributes, and processes used in the simulation, including the decision-making logic of agents, network structures, media influences, and the disease dynamics. The appendix also covers technical implementation details.
(PDF)

S2 Appendix Additional details on model fitting and extended results This section presents further explanations of our model calibration procedures and includes additional plots that expand on the simulation results shown in the main text. (PDF)

S3 Appendix Calibration parameters: tables of definitions, ranges, and fitted values. This appendix provides the parameter values used in the seven fitted models, organized into comprehensive tables. It includes parameter tuples that reproduce behavioral and epidemiological patterns observed in the calibration datasets.
(PDF)

S4 Appendix COVID-19 vaccine eligibility timelines by age group and U.S. state. The table of dates on which various U.S. states expanded COVID-19 vaccine eligibility to specific age groups. The threshold ages (lower bounds) are specified in the table header. This information reflects the phased approach states adopted to distribute vaccines during the pandemic, prioritizing older populations before gradually including younger age groups.
(PDF)

S5 Appendix Variance-based global sensitivity analysis (Sobol indices) This supporting information provides additional results from the variance-based global sensitivity analysis conducted using Sobol indices which quantifies how uncertainty in model parameters contributes to variability in model outcomes.
(PDF)

S6 Appendix Supplementary scenario without media influence, NPI mandates, or vaccine introduction. This supporting information provides additional results for a scenario without media influence, NPI mandates, and vaccine introduction, designed to explore the scenario where opinion dynamics driven solely by peer interactions.
(PDF)
