# Supplementary material for: Modeling individual self-protective behavior during epidemics
Source: PLoS Comput Biol. 2026 May 8;22(5):e1014252. doi: 10.1371/journal.pcbi.1014252 (PMC13170966; doi:10.1371/journal.pcbi.1014252)
Supplement: S1 Appendix — This appendix provides a comprehensive description of our agent-based model following the ODD (Overview, Design concepts, Details) protocol. It details the entities, attributes, and processes used in the simulation, including the decision-making logic of agents, network structures, media influences, and the disease dynamics. The appendix also covers technical implementation details. (PDF) [file pcbi.1014252.s001.pdf]

S1 Appendix. Detailed model description following the ODD protocol

This appendix presents the agent-based simulation model that we proposed following the ODD (Overview, Design concepts, Details) protocol, which was developed to standardize the description of complex agent-based simulation models [1]. To minimize redundancy, we avoid repeating content already explained in the main article. When necessary, we refer to relevant sections, figures, or tables in the main article

The simulator described in this document was written in C++ and compiled using the GNU C++ compiler. Other programming tools, including Python and R, are used to prepare the pre-generated input files that characterize the simulated population. For the model fitting process, functions from SciPy and other Python packages are employed. All simulations were executed on a virtual machine instance hosted on Google Cloud Platform. The instance was configured with the e2-highcpu-16 machine type, which includes 16 virtual CPUs and 16 GB of memory. The simulation environment was based on Debian 12. The instance was used exclusively for computational tasks related to model calibration and scenario-based simulations.

1 Purpose

The model is designed to simulate the spread of an airborne disease within artificial human populations, where each individual's self-protective behavior is determined by their own decision-making process. This process considers factors such as personal perception of the situation, age, and resource availability. Additionally, each individual's perception of disease spread is updated throughout the simulation while influenced by media channels and interactions with their neighbors.

The simulated disease mimics selected characteristics of COVID-19, including human-to-human transmission through close contact, an incubation period, waning immunity, and possible reinfection. To implement these traits in our simulator, we adopt the SEIRS model for networked populations.

In this paper, the simulation model serves as a tool to reproduce trends observed during the COVID-19 pandemic among the U.S. population. It is also designed to simulate alternative scenarios that deviate from the baseline testbed identified through the model fitting process. Individual-level decision-making and the collective patterns of such decisions within demographic groups are challenging to observe in real-world pandemic events. By simulating both baseline and alternative scenarios, our model contributes to understanding these behaviors under the assumption that individuals make decisions in a reasonable, self-serving manner, even when their access to information is limited or biased. By incorporating individual-level motivations for self-protective decisions against infectious diseases, we provide novel insights into policy design for disease mitigation.

2 Entities, attributes, and scale

Our agent-based simulation model includes three types of entities: individual agents, media channels (Channel One and Channel Two), and the vaccine administrator entity. The simulated population refers to the set of N individual agents, as they are the primary focus of our study, while the other two types of entities constitute the environment. Individual agents interact with one another through two networks: the opinion network and the contact network, as shown in Fig 1 of the main article.

The agent attributes that correspond to the opinion dynamics, disease dynamics, and decision-making are listed in the following two tables. Table A shows the agent

attributes that are constant throughout a simulation run. In this study, these attributes define the underlying characteristics of the agent's identity, such as whether they belong to a younger or older generation. The simulation population's Deg_k, A_k, and Y_k are generated based on U.S. datasets. On the other hand, the two attributes M_k and F_k, which determine media usage behavior, are generated using beta distributions, with their means and variances later calibrated to reproduce patterns observed in real-world datasets. The beta distribution is adopted to ensure that both types of usage values remain within the range $[0, 1]$ and that their distributions take on the desired shapes: for example, a skewed bell shape for unimodal but asymmetrical usage patterns and a U-shape for polarized usage patterns. The opinion network is static over time, and so is the neighbor set of agents, as we assume that people tend to share their ideas only with their close or trusted few [2,3]. We obtain the opinion networks through the Poisson random network generation process [4,5].

Table A. Static attributes of agent k

Notation	Attribute Description	Remarks
M_k	Relative usage of media channels as a source of information ($\in [0, 1]$). That is, agent k receives messages from the two media channels with probability M_k and from its neighbors in the opinion network with probability $(1 - M_k)$.	Opinion dynamics
F_k	Relative usage of the pro-intervention channel ($\in [0, 1]$). That is, agent k receives messages from the pro-intervention channel with probability F_k and from the anti-intervention channel with probability $(1 - F_k)$.	Opinion dynamics
$\mathcal{N}_k^{(O)}$	The set of Agent k's neighbors in the opinion network.	Disease dynamics
Deg_k	The degree of agent k in the contact network, represented as an integer.	Disease Dynamics
A_k	The age of agent k, represented as an integer.	Decision-making
Y_k	The resource availability of agent k, represented as a rational number. These values are generated from an income distribution dataset, used as a proxy for individual resource availability.	Decision-making

Table B lists the attributes of agents that change over time. The first four attributes of perceived probabilities and uncertainty values are updated following the opinion dynamics model. $D_{k,t}$ tracks the disease status of the agent k and $\mathcal{N}_{k,t}^{(C)}$ tracks the set of neighbors in the contact network at time t. It is assumed that the contact network is dynamic, while maintaining the same degree configuration. Network instances are generated by the configuration model keeping the population mix as desired using a modified method [6,7]. The last three attributes keep the agent's self-protective behavior as an output of their own decision-making processes. The specific mechanisms by which individual agents make self-protective decisions against infectious diseases are explained in the next section. Note that by "NPI compliance," we refer to an individual's decision to adhere to government or authority guidelines for self-protection against infectious diseases, regardless of whether they are mandatory or optional. That is, an agent k's attribute $C_{k.t}$ has the value "$True$" if the person wears a mask or maintains social

distancing, regardless of whether such NPIs are mandatory at the moment or not.

Table B. Dynamic attributes of agent k

Notation	Attribute Description	Remarks
$\tilde{P}_{k,t}^{(0,0)}$	The perceived probability of infection when not vaccinated and not complying with NPI guidance ($\in [0,1]$).	Opinion dynamics
$\tilde{P}_{k,t}^{(s)}$	The perceived probability of experiencing side effects when vaccinated ($\in [0,1]$).	Opinion dynamics
$U_{k,t}^{(0,0)}$	Agent k's uncertainty value regarding its current perception of $\tilde{P}_{k,t}^{(0,0)}$.	Opinion dynamics
$U_{k,t}^{(s)}$	Agent k's uncertainty value regarding its current perception of $\tilde{P}_{k,t}^{(s)}$.	Opinion dynamics
$D_{k,t}$	Agent k's disease status at time t ($\in \{S,E,I,R\}$).	Disease dynamics
$\mathcal{N}_{k,t}^{(C)}$	The set of Agent k's neighbors in the contact network.	Disease dynamics
$C_{k,t}$	Agent k's NPI compliance behavior at time t, represented as a Boolean value.	Decision-making
$W_{k,t}$	Agent k's vaccination willingness status at time t, represented as a Boolean value.	Decision-making
$V_{k,t}$	Vaccination status of agent k at time t as a Boolean value: $True$ if vaccinated by time t, $False$ otherwise.	Decision-making

The initialization process for these attributes is explained later in this document.

Our model features two media channels: one representing real-world media sources that broadcast pro-intervention messages and the other representing channels that promote anti-intervention messages. These two channels are designed to primarily influence opinion dynamics; however, individual agents can also update or reinforce each other's opinions through interactions. Initially, both media entities broadcast messages about the infectiousness of the disease. Once vaccine administration begins, their messaging shifts to focus on the vaccine's side effects.

As components of the opinion dynamics model, these media channels are characterized by fixed opinion and uncertainty values throughout each simulation run, akin to stubborn agents or opinion leaders in existing opinion dynamics studies [8–12]. Pro-intervention messages assert a higher perceived infectiousness of the disease ($P_{\text{channel 1}}^{(0,0)}$) and a lower perceived risk of vaccine side effects ($P_{\text{channel 1}}^{(s)}$). Conversely, anti-intervention messages assert a lower perceived infectiousness of the disease ($P_{\text{channel 2}}^{(0,0)}$) and a higher perceived risk of vaccine side effects ($P_{\text{channel 2}}^{(s)}$). Channel One's messages are relatively more inclined to encourage people to comply with public health authorities interventions, such as adhering to NPI guidelines and getting vaccinated, by increasing individual agents' concern about the infectiousness of the disease and reducing their concern about the vaccine side effects. In contrast, Channel Two's messages are inclined to promote the opposite behavior.

The following table summarizes the attributes assigned to these two media channel entities.

Table C. Media channel parameters.

Description	Channel One	Channel Two
Channel tendency	Pro-intervention	Anti-intervention
These values represent the perceived probabilities of infection that an individual would experience if they are unvaccinated and do not adhere to the NPI guidelines and that each channel intends to convey to its audience.	$\tilde{P}^{(0,0)}_{\text{channel 1}} = 0.25$ (high)	$\tilde{P}^{(0,0)}_{\text{channel 2}} = 0.00$ (low)
These values represent the uncertainty of the corresponding probability ($\tilde{P}^{(0,0)}_{\text{channel 1}}$ and $\tilde{P}^{(0,0)}_{\text{channel 2}}$). A lower uncertainty value reflects stronger persuasive power or a stronger influence.	$U^{(0,0)}_{\text{channel 1}} = 0.05$ (fixed)	$U^{(0,0)}_{\text{channel 2}} \in [0.05, 0.3]$
These values represent the perceived probabilities of experiencing side effects when vaccinated and that each channel intends to convey to its audience.	$\tilde{P}^{(s)}_{\text{channel 1}} = 0.00$ (low)	$\tilde{P}^{(s)}_{\text{channel 2}} = 0.75$ (high)
These values represent the uncertainty of the corresponding probability ($\tilde{P}^{(s)}_{\text{channel 1}}$ and $\tilde{P}^{(s)}_{\text{channel 2}}$). A lower uncertainty value reflects stronger persuasive power or a stronger influence.	$U^{(s)}_{\text{channel 1}} = 0.05$ (fixed)	$U^{(s)}_{\text{channel 2}} \in [0.05, 0.3]$

The vaccine administrator entity is responsible for changing the vaccination status ($V_{k,t}$) of willing agents ($W_{k,t} = \text{True}$) from $False$ to $True$. Its administration capacity is initially zero, as no vaccine is available at the beginning. The capacity changes to $\mathcal{K}_1$ and $\mathcal{K}_2$ over time as the vaccine becomes available and the administration capacity expands. The administrator also determines whether an agent is eligible for vaccination. Details on how this administrator operates under each eligibility rule are explained in the submodel section of this document.

The population size (N), the main scale of our model, is set to 20,000, meaning that we simulate interactions between 20,000 individual agents and observe the resulting dynamics. This population size is strategically chosen for the following reasons. First, it is large enough to prevent the early extinction of the infectious disease due to a small population combined with the stochasticity of our model. Unlike deterministic compartmental models, agent-based simulations with stochastic disease transmission cannot have fractional numbers of new infections. Therefore, if the population size were too small, there would be a high risk of early disease extinction in the initial stages of the simulation. At the same time, we cannot increase the population size too much due to limitations in the computational resources available for our study.

Our model does not incorporate any geospatial characteristics. Interactions between agents are solely defined by the networks connecting them, which are derived from the age-group contact matrix. It operates under the assumption that the population is a single large, well-mixed group, without spatial or community-level segregation.

3 Process overview and scheduling

For each simulation instance, the following submodels are executed in order, iteratively, after initialization and continue until the end of the instance: 1) environment update module, 2) opinion dynamics module, 3) vaccination module, 4) NPI compliance module, and 5) disease dynamics module. The overall flow is also illustrated in Fig A, and the detailed operations of each module are explained in the following submodels section. After completing all iterations, the program records the output to JSON files.

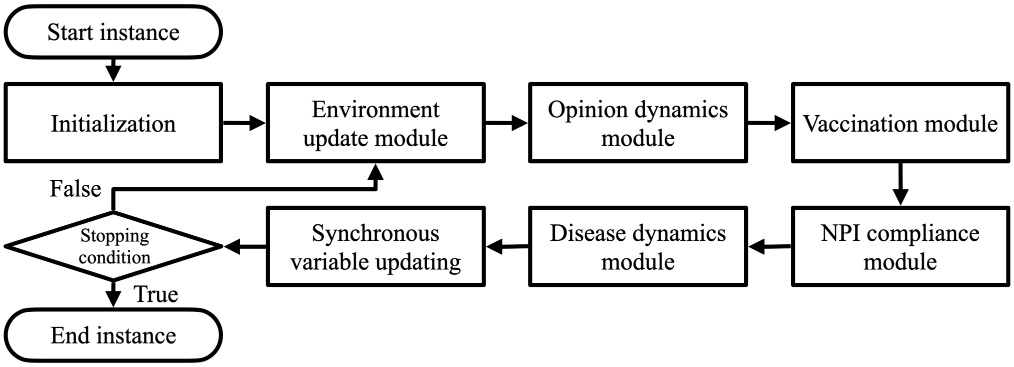

Fig A. Simulation procedure flow for a single instance.

All the dynamic attributes and variables that may change over time are updated at the end of the corresponding modules' operations or updated synchronously for each module, following the synchronous activation regime [13]. For example, attributes related to the opinion dynamics (e.g., $\tilde{P}^{(s)}_{k,t}$) of all agents are updated synchronously at the end of the opinion dynamics module. Our model follows discrete time steps, assuming time moves forward in days. When the simulation time reaches day 450, the stopping condition for the simulation run is considered satisfied, and the instance ends.

We note that the model adopts a one-directional structure in which disease dynamics do not feed back into opinion formation or behavioral decision-making.

4 Design concepts

4.1 Basic principles

The basic design principles are explained in the "Model and Methods" section of our main manuscript.

4.2 Emergence

The collective patterns of agents' NPI compliance and vaccination, particularly within each demographic subgroup, are emergent properties of our simulation model. The patterns of the resulting disease dynamics are also a key emergence of our model.

4.3 Adaptation and objectives

Individual agents update their perception of the spreading disease and the level of certainty they have about their perception values. The opinion dynamics module governs how this adaptation occurs throughout a simulation run. Based on their attribute values, individual agents also make decisions regarding whether they will comply with the NPI guidelines and whether they are willing to get vaccinated. The NPI compliance module and the vaccination module facilitate this adaptation. Following is the detailed description about the decision tree we designed for our study.

Individual agents make decisions at each time step regarding whether they will comply with the NPI guidance, such as wearing a mask or maintaining social distancing, and whether they are willing to get vaccinated if they have not already been vaccinated. The decision tree method is used to model these decision-making processes for individual agents.

In this study, it is assumed that each agent makes decisions using the three decision trees shown in Fig B, based on their own opinions, to maximize their expected utility. To represent individual agents' decision-making regarding vaccination and NPI compliance, we adopt a structured decision framework in the form of a sequential decision tree [14, 15]. The sequential decision tree is composed of decision nodes and chance nodes. At decision nodes (squares), agents compare the expected values of available actions and select the branch with the highest value. At chance nodes (circles), uncertainty is represented by computing a weighted average of downstream branch values according to their associated probabilities. The computation proceeds backward from the leaf nodes to the root, ensuring that decisions at each node are evaluated based on the expected values of their subsequent outcomes. The final output of the decision tree is the decision at the root node that is corresponding to the branch with the largest assigned value.

Trees (A1) and (A2) are used to make decisions regarding an agent's NPI compliance, while tree (B) is used to determine their vaccination willingness at time t. In the superscript of the probability values of the decision trees, there are four different behavioral combination codes: (0,0), (0,1), (1,0), and (1,1). The first value represents the behavior of complying with NPIs, and the second value represents their vaccination status. For example, (1,0) represents the case where an agent is complying with NPI while not vaccinated. The probability values with these superscripts represent the perceived probability of getting infected, which agents use to assess their options. Note that only two probability values are considered as an agent's perception that changes over time, $\tilde{P}_{k,t}^{(0,0)}$ and $\tilde{P}_{k,t}^{(s)}$, while other probability values are fixed. This is to minimize the complexity of the model and have a single dynamic opinion value closely related to each of the two self-protection behaviors: NPI compliance and vaccination willingness. These two values represent the risk of infectious disease without any self-protection measure ($\tilde{P}_{k,t}^{(0,0)}$) and the risk of vaccine's side effects that are perceived by an individual ($\tilde{P}_{k,t}^{(s)}$), respectively. Since these probability values in the tree are values that each agent uses to make their own decisions, they do not convey the exact characteristics of the disease.

In the branches where an agent becomes infectious, or the branches labeled with one of $P^{(0,1)}$, $P^{(1,1)}$, $P^{(1,0)}$, and $\tilde{P}_{k,t}^{(0,0)}$, the agent's resource availability (Y_k) is adjusted with the modifier α_k. We assume that this modifier reduces the agent's resource availability mildly if its age value is smaller than 65 ($\alpha_k = \alpha_k^{<65}$) and largely if the agent is in the 65+ age group ($\alpha_k = \alpha_k^{65+}$). The branches labeled with $\tilde{P}_{k,t}^{(s)}$ in Tree (B) represent the case where an agent is getting vaccinated and experiences severe side effects. A similar reduction to the agent's resource availability is assigned to those cases with ξ_k, which also affects the agent's resource level differently based on its age.

For the cases in which agents decide to comply with non-pharmaceutical interventions, the value of $C^{(n)}$ is deducted from their baseline resource availability. This can be interpreted as any difficulties and inconveniences that agents experience while complying with NPIs or monetary costs for medical supplies that are required for such compliance. If an agent decides not to comply with NPIs, then the penalty ($C^{(p)}$) can be imposed on those agents, depending on the government's NPI policy at the moment. This can be interpreted as penalties and inconveniences that someone who does not comply with the NPI mandate would experience, such as a ban on entering

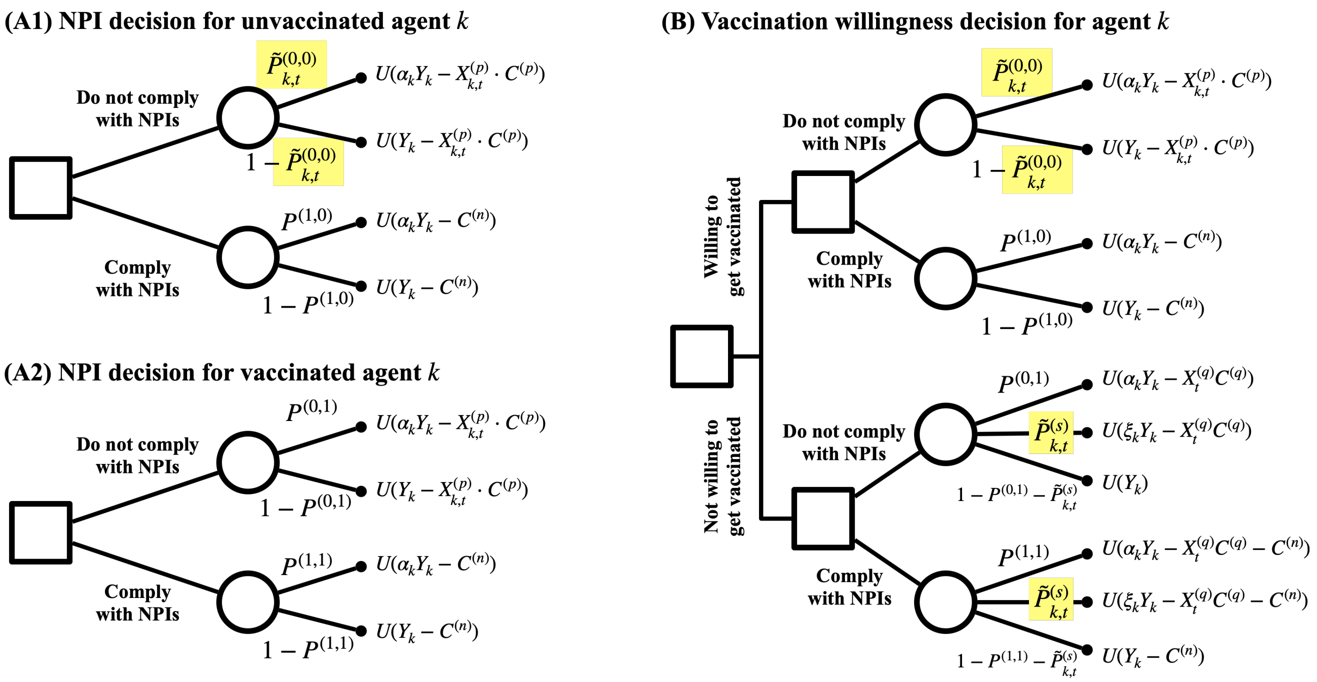

Fig B. Decision trees for individual agents. Individual agents make decisions through three decision trees. An agent's compliance with NPIs, such as wearing a mask or maintaining social distancing, is determined through decision trees (A1) and (A2), depending on its vaccination status. If an agent is eligible for vaccination, it decides on its willingness to get vaccinated using the decision tree (B). Only two probability values (highlighted) are based on an agent's perception that changes over time: $\tilde{P}_{k,t}^{(0,0)}$ and $\tilde{P}_{k,t}^{(s)}$, while other probability values are fixed at a certain value for simplification. This is to minimize the complexity of the model and have only one dynamic opinion value closely related to each of the two self-protection behaviors: NPI compliance and vaccination willingness.

buildings, social pressure caused by neighbors' judgment, or an actual fine that they may need to pay. If an NPI mandate is imposed on the agent at the time, then $X_{k,t}^{(p)}$ is set to 1, and the penalty cost is activated. Lastly, agents in our model tend to be less motivated to get vaccinations when the administration queue is crowded. $C^{(q)}$ is such a cost for going through a crowded vaccination queue. $X^{(q)}$ is used to represent the status of the queue as a boolean variable. Due to this cost, some agents may choose to delay vaccination, particularly when the expected benefit is low relative to the inconvenience.

Our design of the decision tree is inspired by studies on insurance and utility, as self-protection measures against infectious diseases are similar to insurance in the sense that individuals may incur costs to reduce potential losses or the risk of such losses [15]. This model of individual-level decision-making is simplified and obscures many details of the real-world decision-making problem. We use a linear utility function and do not apply temporal discounting for model simplicity; consequently, the output of the utility function is equal to its input. Limitations of this approach are discussed at the end of this article.

Individual agents may or may not be willing to incur various costs to protect their utility from losses caused by infectious diseases. The assumptions and setup of this study suggest an explanation for decisions such as getting vaccinated while not wearing a mask, as some agents in the simulation may perceive "double protection" through both vaccination and NPI compliance as too costly, depending on their personal opinions and circumstances. The relative relationships between costs and resource reduction rates play a dominant role in shaping NPI compliance and vaccination

decisions, because notions such as “more” or “less” expensive depend on this relativity.

4.4 Learning and prediction

Unlike adaptation, learning, defined as making changes over time based on an agent’s past experiences, is not incorporated into this model. Although we use the term “learning rate” as part of the relative agreement model when updating agents’ opinions and uncertainty, this term specifically refers to the extent to which changes are applied to agents’ previous attribute values. It does not imply that agents are learning from past experiences, behaviors, or decisions.

Prediction is also not a component of this simulation model, as agents are not designed to make long-term or persistent impacts through their decision-making. Instead, they consider a given set of parameter values and make decisions based solely on these inputs. Notably, decision-making in our model may reflect long-term cumulative costs, as we use annual income as a measure of agents’ resource availability and set NPI compliance costs within the range of the annual supply cost of face masks. However, the simulation mechanism itself does not specifically endow agents with predictive capabilities.

4.5 Sensing

First, individual agents are notified whether the NPI remains a guideline or is imposed as a mandate. Next, once vaccine administration begins at t_3, agents can check their eligibility for vaccination. Also, if an agent is willing to get vaccinated, they can also determine whether they will receive the vaccine at the current time step. This means there are no cases where an agent intends to get vaccinated but fails due to external constraints, such as limited supply or long wait times, after waiting in the queue. If an agent is unable to get vaccinated due to a capacity limit, the agent will not incur any additional costs.

4.6 Interaction

All the interactions between agents are defined by the two networks: the opinion network and the contact network. As overviewed in Fig 1 of the main article, agents’ opinions are updated through interactions in the opinion network layer via the relative agreement model. The infectious disease spreads throughout the contact network at the same time. The opinion dynamics model and the disease dynamics model are explained in detail in the Submodel section of this document.

4.7 Stochasticity

The state transitions between disease statuses, following the SEIRS model, are stochastic, with assigned probabilities for each transition. The subset of the population that is set as infectious at the beginning of the simulation is also randomly selected. The vaccine administration process is stochastic as well, randomly selecting agents from those who are willing and eligible if its administration capacity cannot vaccinate all such agents. The details of the randomness are explained in later sections for the corresponding modules.

4.8 Collectives

There are no collectives of agents in the model; that is, we do not implement social structures such as families, schools, or workplaces.

4.9 Observation

All the simulator results are saved as JSON files. The following three observations are tracked for both analysis and model calibration purposes:

1. The average NPI compliance levels, recorded over time.
2. The proportion of the vaccinated population, recorded over time.
3. The disease vector, $< |S_t|, |E_t|, |I_t|, |R_t| >$, recorded over time.
4. The number of new infections, recorded over time.

The following observations are recorded for analysis purposes:

5. The proportion of the vaccinated population over time among the 65+ population.
6. The number of new infections over time among the 65+ population.
7. The NPI compliance for each decile group in three different ordering criteria, recorded over time.
8. The proportion of the vaccinated population for each decile group in three different ordering criteria, recorded over time.
9. The number of new infections for each decile group in three different ordering criteria, recorded over time.
10. The number of pressured compliance for each decile group in three different ordering criteria, recorded over time.

The three ordering criteria refer to age, resource availability, and degree in the contact network.

4.10 Initialization and inputs

In this section, we first discuss how the simulation initialization is conducted and then describe the input files to the model. Most of the input files, which are generated in advance, are loaded into the simulator at the instance initialization stage, except for the contact networks, which are loaded newly at every time step of a simulation run.

In the initialization stage for a simulation instance, we first generate an artificial population of 20,000 individual agents. The population's attribute values that change over time are initialized by loading the following input files.

The first input the simulator loads is a vector of agents' age and resource availability pairs, $\langle (A_k, Y_k) \text{ for } k \in \{1, 2, \dots, N\} \rangle$. The input files in this category are pre-generated based on the age distribution of the US population in 2019 and the joint distribution of age and income of the US population in 2019. Fig 4(a) of the main article visualizes the input data of the joint distribution.

The next input is the vector of the simulated population's media usage, $\langle M_k \text{ for } k \in 1, 2, \dots, N \rangle$. M_k represents the probability that agent k receives messages from the two media channels at each time step. That is, the agent receives messages from its neighbors in the opinion network with probability $(1 - M_k)$. We use the beta distribution to generate the values. This is to ensure that all the usage values fall within the range of $[0, 1]$. Since the relative preference for mass media compared to one's opinion-sharing neighbors as a source of information is beyond the scope of our research, we fixed the distribution to be centered at 50% and symmetric with a bell-shaped curve. We set the mean and variance of the distribution to 0.5 and 0.01. Note that the

complex nature of people's preferences in media consumption invited multiple studies and ongoing discussions [16–19].

The input vector of the relative channel usage values, $\langle F_k \text{ for } k \in 1, 2, \ldots, N \rangle$, is loaded next. Our model has two mass media channels: one with pro-intervention messaging and the other with anti-intervention messaging, which drive the opinion dynamics together while competing with each other. F_k denotes the agent's probability of receiving messages from the pro-intervention channel. That is, it receives messages from the anti-intervention channel with probability $(1 - F_k)$. Again, the distribution follows a beta distribution with two given shape parameters: mean and variance. Based on these two parameters, we can generate patterns indicating polarization between two opposing preferences [20]. Note that beta distributions are well-suited to model bell-shaped and U-shaped patterns, which are considered typical patterns of opinion distribution in existing studies [21–23]. These two parameters are treated as free parameters for model fitting, as we are manipulating the opinion dynamics to fit the NPI compliance patterns to the calibration data.

The two vectors of the initial values of $\tilde{P}^{(0,0)}_{k,t=0}$ and $\tilde{P}^{(S)}_{k,t=0}$ are also loaded. As before, the values need to be contained within the range of $[0, 1]$ because they serve as probabilities. For this reason, beta distributions are utilized to generate the input vectors. These two parameters are also treated as free parameters for model fitting. This is because these values are important for reproducing the early patterns of NPI compliance and vaccination behaviors, as they determine the starting point of such patterns. The corresponding uncertainty values for these perceived probabilities of agents are also initialized at the same time. We assume that all the agents in the system have homogeneous initial uncertainty for each, following the uncertainty initialization for regular agents similar to the existing study's setup [8].

The opinion network and the contact network are also loaded into the simulator as inputs. We obtain the opinion networks through the Poisson random network generation process, which is widely accepted in networked modeling studies, and the contact networks using a configuration model while maintaining the desired population mix through a modified method. The population mix is visualized in Fig 4(b) of the main article.

Finally, we initialize the infectious population by selecting a specified number of agents. We specifically select a set of agents whose average age, resource availability, and contact degree closely approximate the overall population averages, with a maximum deviation of 1%. This method aims to ensure that the initial conditions of the epidemic are representative of the population's average characteristics, thereby enhancing the model's relevance and reducing the volatility of simulation outcomes.

5 Submodels

In this section, we describe the details of modules listed in the simulation process flow in Fig 2 of the main article. The order of modules explained also follows the procedure flow.

5.1 Environment update module

The environment update module updates the entity attributes and variables related to the environment. By "environment," we refer to factors that globally affect all individual agents and their decision-making.

The NPI guideline and mandate. First, the policy regarding nonpharmaceutical interventions (NPI) changes over time. Initially, it remains a guideline or recommendation until time t_1, when it becomes mandatory to follow the NPI guidance. Under the mandate, a penalty value $(C^{(p)})$ is imposed on those who do not comply. The

binary variable $X_{k,t}^{(p)}$ determines whether the penalty value is imposed on agent k at each time step t. The following equation describes how this binary variable is assigned its value in this module.

$$X_{k,t}^{(p)} = \begin{cases} 0, & \text{if } t < t_1 \quad \text{(NPI mandate not applied yet)} \\ & \text{or } (t \geq t_2 \text{ and } V_{k,t} = 1) \quad \text{(NPI mandate lifted, agent } k \text{ is vaccinated)} \\ \\ 1, & \text{if } t_1 \leq t < t_2 \quad \text{(NPI mandate in effect)} \\ & \text{or } (t \geq t_2 \text{ and } V_{k,t} = 0) \quad \text{(NPI mandate lifted, agent } k \text{ is not vaccinated)} \end{cases} \tag{1}$$

Content of the messages from the two media channels. The content of the messages from the two media channels, Channel One and Channel Two, also changes over time. Individual agents share opinions on corresponding topics as the media channel content evolves. Their messages are about the perceived probabilities of infection that an individual would experience if they are unvaccinated and do not adhere to the NPI guidelines before the vaccine introduction ($t < t_3$). Messages then shift to focus on the perceived probabilities of experiencing side effects when vaccinated after the vaccine introduction ($t \geq t_3$).

While discussions about vaccine safety in the United States began before the vaccine's official introduction, we assume in our simulation model that these discussions align with the vaccine introduction date. This assumption reflects our limited knowledge of when the U.S. public actively began assessing vaccine risks based on media coverage. Our model also assumes that the previous opinion topic is no longer updated after t_3. The impact of this assumption is marginal because the uncertainty of individual agents naturally decreases over time under the relative agreement model, and people become less likely to change their opinions as the opinion dynamics progress. However, under alternative opinion dynamics models that allow opinions to fluctuate or shift substantially even after an apparent stabilization, it may be necessary to retain messaging on the initial topic throughout the remainder of the simulation.

Vaccine administration capacity. The vaccine administration capacity ($\mathcal{K}$) is another environment variable governed by this module. This variable determines the maximum number of vaccine administrations that can be processed in a day at time step t as follows:

$$\mathcal{K} = \begin{cases} 0, & \text{if } t < t_3 \\ \mathcal{K}_1, & \text{if } t_3 \leq t < t_4 \\ \mathcal{K}_2, & \text{if } t \geq t_4 \end{cases} \tag{2}$$

Vaccine eligibility threshold. If vaccine eligibility management is conducted on a rolling basis over time, the eligibility threshold is updated accordingly as the thresholds change. The baseline scenario follows an age-based rolling eligibility system. For this study, we referenced the vaccine eligibility timelines of U.S. states during COVID-19 and found that eligibility gradually expanded to include everyone over time (See Table S10). For open eligibility, where every agent is eligible throughout the entire simulation period, this part is not utilized.

Since eligibility expansion patterns were highly heterogeneous across U.S. states, we generated an artificial schedule, shown in Table D, assuming that the proportion of the eligible population grows linearly over time while matched with the introduction date and the time the eligibility is expanded to everyone. The actual schedule's eligibility expansion is slower than the artificial schedule in general. You can find the actual schedules of age-based eligibility in U.S. states during COVID-19 in S4 Appendix.

For the two alternative vaccine eligibility rules, we assumed the same linear expansion with a similar number of changes, as shown in Table E and Table F. This

ensures that we are analyzing the impact of the ordering criteria rather than the effect of varying numbers of eligible agents at a given time. Fig C visualizes the proportions of eligible agents within the total population over time under different rules.

Table D. Age-based rolling eligibility schedule

Time step	255	271	279	287	294	302	310	318	326	341	363
Age (lower bound)	80	75	70	65	60	55	50	45	40	30	15

Table E. Degree-based rolling eligibility schedule

Time step	255	271	279	287	294	302	310	318	326	341	363
Network degree (lower bound)	18	17	15	14	13	12	11	10	10	8	3

Table F. Resource-based rolling eligibility schedule

Time step	275	279	287	294	302	310	318	326	341	363
Resource (upper bound)	1	1172	6788	13724	19332	25230	31973	39438	62188	198750

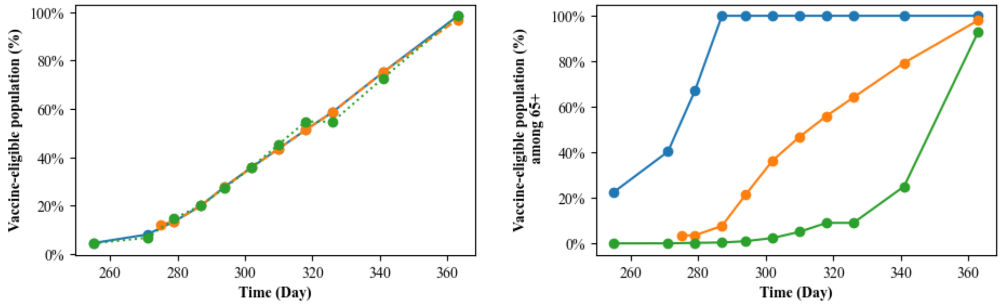

Fig C. The trend of vaccine-eligible population's proportion in the entire population determined by the artificial eligibility schedule used in our study. All three rolling eligibility schedules are designed to expand the eligible population linearly while aligning with the vaccine introduction date and the date when eligibility was expanded to include everyone in the U.S. The second plot illustrates the resulting eligibility trend for the 65+ population. Eligibility in the 65+ population expands the fastest under the age-based schedule and the slowest under the schedule based on contact network degree.

5.2 Opinion dynamics module

This module updates the two opinion values of agents, $\tilde{P}_{k,t}^{(0,0)}$ and $\tilde{P}_{k,t}^{(s)}$, along with their corresponding uncertainty values, $U_{k,t}^{(0,0)}$ and $U_{k,t}^{(s)}$, following the updating rules proposed in the relative agreement model [8].

Each individual agent first randomly selects a source of information to update their opinion. The two attributes, M_k and F_k determine which channels or individual agents will be selected. With probability $(M_k \cdot F_k)$, the source of information is the pro-intervention media channel, while with probability $(M_k \cdot (1 - F_k))$, it is the anti-intervention media channel. With probability $(1 - M_k)$, agent k chooses to learn from its neighbors in the opinion network instead of the media channels. Since our opinion network is unweighted and undirected, the agent selects a neighbor uniformly at random from its set of neighbors. In the following opinion-updating equations, P_{ext} and U_{ext} denote the opinion and the corresponding uncertainty value of the selected entity.

Equations 3 show how the opinion updating rules are defined in the context where the Ps denote the opinion and Us denote the corresponding uncertainties. Equations 3 is used for updating before t_3, and the same equation with the superscripts changed ((s) instead of $(0,0)$) is used starting from t_3 as the given environment changes.

$$\tilde{P}_{k,t+1}^{(0,0)} = \tilde{P}_{k,t}^{(0,0)} + \mu_1^{(0,0)}(h/U_{\text{ext}}^{(0,0)} - 1)(P_{\text{ext}}^{(0,0)} - \tilde{P}_{k,t}^{(0,0)}) \tag{3a}$$

$$U_{k,t+1}^{(0,0)} = U_{k,t}^{(0,0)} + \mu_2^{(0,0)}(h/U_{\text{ext}}^{(0,0)} - 1)(U_{\text{ext}}^{(0,0)} - U_{k,t}^{(0,0)}) \tag{3b}$$

$$\text{where} \quad h = \min(U_{k,t}^{(0,0)}, U_{\text{ext}}^{(0,0)} + |\tilde{P}_{k,t}^{(0,0)} - P_{\text{ext}}^{(0,0)}|) \tag{3c}$$

$$- \max(-U_{k,t}^{(0,0)}, |\tilde{P}_{k,t}^{(0,0)} - P_{\text{ext}}^{(0,0)}| - U_{\text{ext}}^{(0,0)}) \tag{3d}$$

h represents the overlap between the opinion range segments of agent k and the selected external source, where the segments are defined by placing their opinion values at the center and determining their width using the corresponding uncertainty values. The μs are free-range parameters that will also be fitted during the model-fitting steps. These values represent the learning rate, or how quickly agent k's values approach the target values of the learning source in one update operation.

One key property of the RA model [8] is that, under fixed interaction rules and information environments, agent opinions evolve through repeated interactions and eventually converge to a stable configuration. In our calibrated model, opinions associated with perceived disease infectiousness, represented by $\tilde{P}_{k,t}^{(0,0)}$, converge well before the introduction of vaccination at time t_3. After this point, both the opinion values and their associated uncertainties remain effectively stable in the absence of structural changes to the information environment. Although agents' behaviors may continue to change after t_3, the underlying infectiousness-related beliefs no longer meaningfully evolve under the RA dynamics. Consequently, continuing to broadcast media messages targeting infectiousness after vaccine roll-out does not affect agent opinions or downstream behaviors in our simulations. For this reason, once vaccination becomes available, media broadcasts are restricted to vaccination-related messaging ($\tilde{P}_{k,t}^{(s)}$).

We note that this modeling choice is specific to the RA framework and its convergence properties. Under alternative opinion dynamics models—particularly those that allow persistent opinion fluctuations after one convergence, broadcasting of multiple message types could remain influential. Our framework can accommodate such extensions, but they are beyond the scope of the present study.

Vaccination and NPI compliance modules

First, all the vaccine-eligible agents determine whether they are willing to get vaccinated or not and determine their attribute $W_{k,t}(\in \{True, False\})$ by using the decision tree (B) in Fig 3 of the main article starting from t_3 when the vaccine administration is started. Before the introduction of the vaccine to the system ($t < t_3$), the simulation process skips this entire module.

The agents who are willing to put up with the queue congestion are processed with higher priority, and the ones who are not willing enough to do so are processed next. By adding this step, we add one step that distinguishes agents with higher willingness. This step is implemented by the vaccine administrator entity considering agents with $W_{k,t}|(X_t^{(q)} = True) = True$, first. If the number of such agents exceeds the administration capacity, then the administrator uses the random selection to proceed. If the administrator has its capacity not completely utilized yet, then it considers agents with $W_{k,t}|(X_t^{(q)} = False) = True$. Again, random selection is used if there are willing agents more than the vaccine administrator's capacity.

Note that all the entities in the system only consider if agents are vaccinated or not. That is, agents do not know if they are exposed to the disease or are naturally immune to the infectious disease due to their past infection. Also, all the vaccinated agents have their disease status $D_{k,t}$ updated to "Recovered". We assume that every individual agent who is infectious at a certain time point recognizes their symptoms and thus does not try to get vaccinated.

The NPI compliance module determines whether each agent will comply with the NPI as guidance or not ($C_{k,t}(\in \{True, False\})$). Agents use decision trees (A1) and (A2) in Fig 3 of the main article.

5.3 Disease dynamics module

In this module, we determine each agent's disease status for the next time step ($D_{i,t+1}$) based on its current status $D_{i,t}$ and other given conditions. First, we update the contact network to determine the potential path of disease spread. Our model assumes a dynamic contact network while maintaining the same degree of configuration and the relative contact frequency between different age groups. We simply use the independently generated networks for different time steps with the same given configuration to introduce dynamic characteristics of connections.

Next, we update the agents' status of disease. Fig D visualizes the transition between paths between the four different disease statuses that an agent can have. Each agent that is classified as one of each group, $[S]_t$, $[E]_t$, $[I]_t$, and $[R]_t$ changes its disease state following the arrow with the corresponding probability, where $[A]_t$ denotes the set of agents that are in the disease state of A at time t. For example, each agent i in "*Susceptible*" changes its disease status to "*Exposed*" with probability $\lambda(i, j, t)$ for each contact edge ($(i, j) \in [SI]_t$) with its infectious neighbor, where $[SI]_t$ denotes the set of contact edges between susceptible agents ($[S]_t$) and infectious agents ($[I]_t$) at time t.

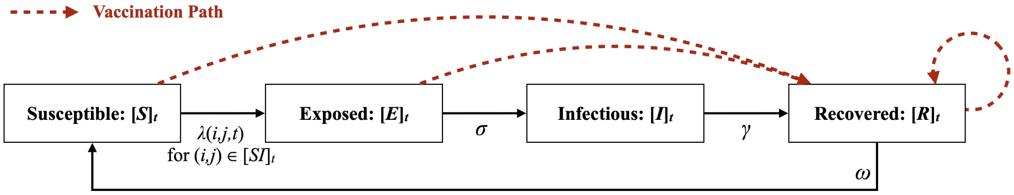

Fig D. Modified SEIRS model used in disease dynamics module.

Note that we assume compliance with NPI guidance reduces the transmission rate if at least one of the two interacting agents follows the guidance [24, 25]. Specifically, if either of the two individuals in contact wears a face mask, the reduced transmission rate is applied to that interaction. Conversely, if neither complies with the guidance, the original transmission rate, λ_0, is used. The following equation describes how this rule is implemented in our simulator:

$$\lambda_{ijt} = \begin{cases} (1-\rho)\lambda_0, & \text{if } \; C_{i,t} = True \; \text{ or } \; C_{j,t} = True \\ \lambda_0 & \text{otherwise.} \end{cases} \tag{4}$$

where ρ represents the reduction rate provided by self-protection measures.

Our model also incorporates external changes in disease spread to account for the first peak in new cases. Multiple studies have discussed various external factors around the first peak of the COVID-19 pandemic, including changes in intervention policies (e.g., restriction change) and shifts in people's behavior (e.g., vacation, holiday seasons, and large political gatherings and rallies before the U.S. presidential election) [26–29]. To incorporate such temporary changes in a simplified manner, our model allows for

adjustments in the intervention's effectiveness (ρ) during a specified period ($[t_a, t_b]$) by a factor of $(1-\delta)$, representing reduced effectiveness.

References

1. Grimm V, Berger U, DeAngelis DL, Polhill JG, Giske J, Railsback SF. The ODD protocol: a review and first update. Ecological Modelling. 2010;221(23):2760–2768.

2. Page-Tan C, Marion S, Aldrich DP. Information trust falls: The role of social networks and information during the COVID-19 pandemic among suburbanites. RSF: The Russell Sage Foundation Journal of the Social Sciences. 2022;8(8):32–51.

3. Wu WL, Lin CH, Hsu BF, Yeh RS. Interpersonal trust and knowledge sharing: Moderating effects of individual altruism and a social interaction environment. Social Behavior and Personality: an international journal. 2009;37(1):83–93.

4. Bansal S, Grenfell BT, Meyers LA. When individual behaviour matters: homogeneous and network models in epidemiology. Journal of the Royal Society Interface. 2007;4(16):879–891.

5. Volz E. SIR dynamics in random networks with heterogeneous connectivity. Journal of Mathematical Biology. 2008;56:293–310.

6. Newman ME, Strogatz SH, Watts DJ. Random graphs with arbitrary degree distributions and their applications. Physical Review E. 2001;64(2):026118.

7. Talaga S, Nowak A. Homophily as a Process Generating Social Networks: Insights from Social Distance Attachment Model. Journal of Artificial Societies and Social Simulation. 2020;23(2). doi:10.18564/jasss.4252.

8. Deffuant G, Amblard F, Weisbuch G, Faure T. How can extremism prevail? A study based on the relative agreement interaction model. Journal of Artificial Societies & Social Simulation. 2002;5(4).

9. Deffuant G, Neau D, Amblard F, Weisbuch G. Mixing beliefs among interacting agents. Advances in Complex Systems. 2000;3(01n04):87–98.

10. Friedkin NE, Johnsen EC. Social influence and opinions. Journal of Mathematical Sociology. 1990;15(3-4):193–206.

11. Friedkin NE, Johnsen EC. Social influence network theory: A sociological examination of small group dynamics. vol. 33. Cambridge University Press; 2011.

12. Shang Y. An agent based model for opinion dynamics with random confidence threshold. Communications in Nonlinear Science and Numerical Simulation. 2014;19(10):3766–3777.

13. Alizadeh M, Cioffi-Revilla C. Activation regimes in opinion dynamics: comparing asynchronous updating schemes. Available at SSRN 2830325. 2015;.

14. Magee JF. Decision trees for decision making. Harvard Business Review Brighton, MA, USA; 1964.

15. Kaas R, Goovaerts M, Dhaene J, Denuit M. Utility theory and insurance. Modern actuarial risk theory: Using R. 2008; p. 1–16.

16. Perrin A. Social media usage. Pew Research Center. 2015;125:52–68.

17. Auxier B, Anderson M. Social media use in 2021. Pew Research Center. 2021;1:1–4.

18. Poushter J, Bishop C, Chwe H. Social media use continues to rise in developing countries but plateaus across developed ones. Pew research center. 2018;22:2–19.

19. Shearer E, Grieco E. Americans are wary of the role social media sites play in delivering the news. Pew Research Center. 2019;2.

20. Assenova VA. Modeling the diffusion of complex innovations as a process of opinion formation through social networks. PloS ONE. 2018;13(5):e0196699.

21. Golbeck J, Hansen D. A method for computing political preference among Twitter followers. Social Networks. 2014;36:177–184.

22. Lyu H, Luo J. Understanding Political Polarization on Social Platforms by Jointly Modeling Users, Connections and Multi-modal Post Contents in Heterogeneous Graphs. arXiv preprint arXiv:220105946. 2022;.

23. Waller I, Anderson A. Quantifying social organization and political polarization in online platforms. Nature. 2021;600(7888):264–268.

24. Wang Y, Deng Z, Shi D. How effective is a mask in preventing COVID-19 infection? Medical Devices & Sensors. 2021;4(1):e10163.

25. Yu G, Garee M, Ventresca M, Yih Y. How individuals' opinions influence society's resistance to epidemics: an agent-based model approach. BMC Public Health. 2024;24(1):863.

26. Britton T, Ball F. Summer vacation and COVID-19: effects of metropolitan people going to summer provinces. arXiv preprint arXiv:200600579. 2020;.

27. Glaeser EL, Jin GZ, Leyden BT, Luca M. Learning from deregulation: The asymmetric impact of lockdown and reopening on risky behavior during COVID-19. Journal of regional science. 2021;61(4):696–709.

28. Yamana T, Pei S, Kandula S, Shaman J. Projection of COVID-19 cases and deaths in the US as individual states re-open May 4, 2020. MedRxiv. 2020; p. 2020–05.

29. Bernheim BD, Buchmann N, Freitas-Groff Z, Otero S. The effects of large group meetings on the spread of COVID-19: The case of Trump rallies. In: Nina and Freitas-Groff, Zach and Otero, Sebastián, The Effects of Large Group Meetings on the Spread of COVID-19: The Case of Trump Rallies (October 30, 2020); 2020.
