# Supplementary material for: Modeling individual self-protective behavior during epidemics
Source: PLoS Comput Biol. 2026 May 8;22(5):e1014252. doi: 10.1371/journal.pcbi.1014252 (PMC13170966; doi:10.1371/journal.pcbi.1014252)
Supplement: S2 Appendix — (PDF) [file pcbi.1014252.s002.pdf]

S2 Appendix. Additional details on model fitting and extended results

This supporting information document provides further explanantion about the model fitting and plots offering additional details on the simulation results presented in the main article.

We fitted our model with the following seven setups: $C^{(p)} = 100$, 150, 200, 250, 300, 350, and 400. The trends of NPI compliance levels and vaccination are primarily driven by the relationships between cost-related parameters, as agents make decision to minimize the potential loss by infection. That is, fixing one cost parameter systematically determines the others once model calibration is complete. Since we aim to set up testbeds across systematically distinct parameter sets to avoid using testbeds similar to each other, we set $C^{(p)}$—the penalty cost for non-compliance with the NPI mandate—to the selected values and proceed. Another cost variable, $C^{(n)}$, represents the cost of compliance, which can be interpreted as the sum of annual expenses, such as the price of N95 face masks. These costs are fitted to values ranging between 100 and 500 under the given $C^{(p)}$ and the fitted models. We can claim that these $C^{(n)}$ values are within the acceptable range that can be explained with real-world price values. For example, if we assume the price of a face mask is \$2.50 and a person changes their mask every three days, the annual cost will be approximately \$300. This value can be smaller or larger based on the assumptions of the usage duration or the mask price. The experience of inconvenience as an additional monetary cost value can also be added to $C^{(n)}$ for larger cost values. Fig A and Fig B show the seven calibrated models with different $C^{(p)}$ values used in the aggregation process, highlighting that they are generally similar with minor differences. All the results in the main article are based on the average of these seven fitted models.

Fig C, Fig D, and Fig E present subpopulation patterns in the same format as Fig 7 of the main article.

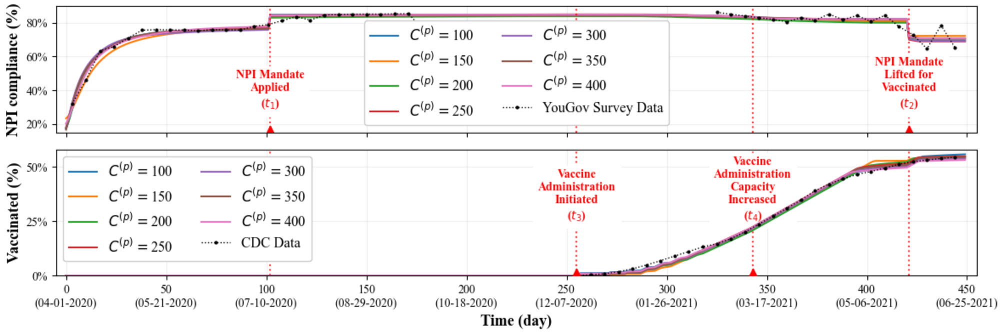

Fig A. Simulation results of the seven baseline models: average NPI compliance level trend over time (top) and trend (%) of the vaccinated population (bottom). The average of these results are shown in Figure 3. of the main article.

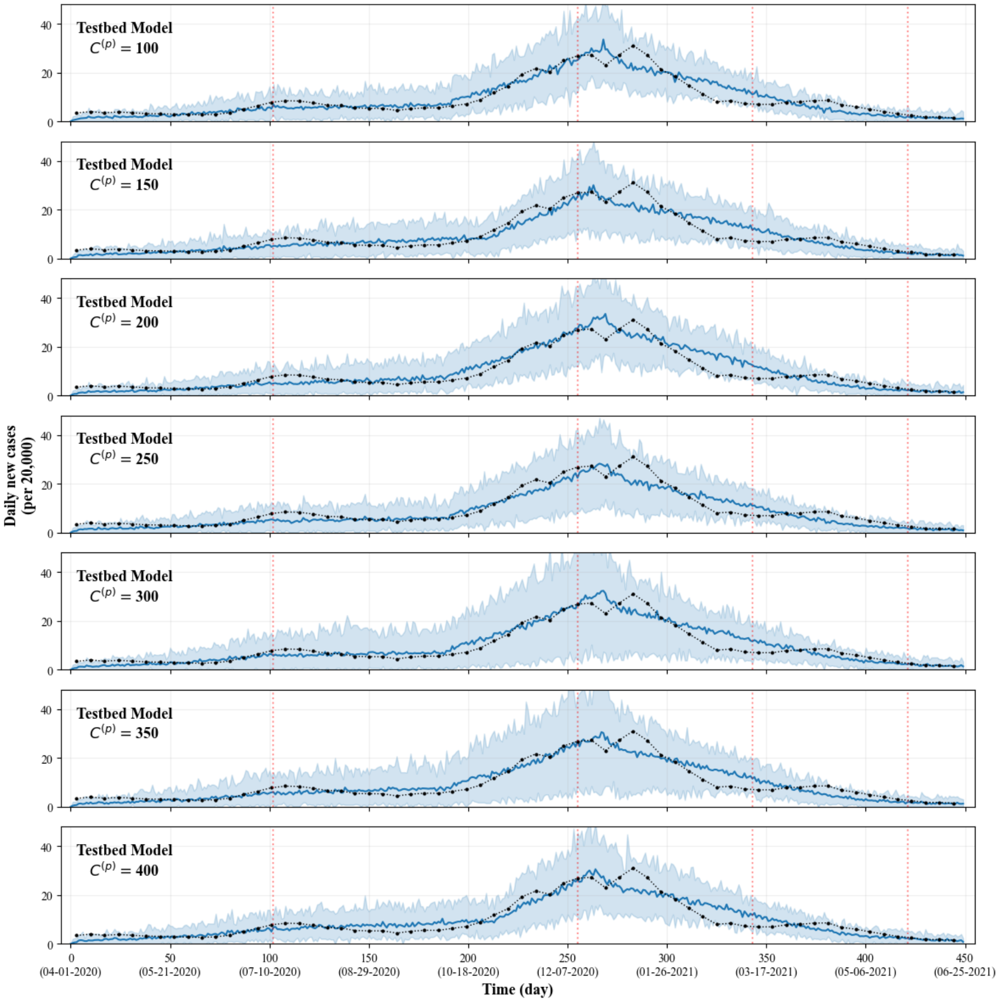

Fig B. Simulation results of the seven baseline models: daily new case trend. The average of these results are shown in Fig 5. of the main article.

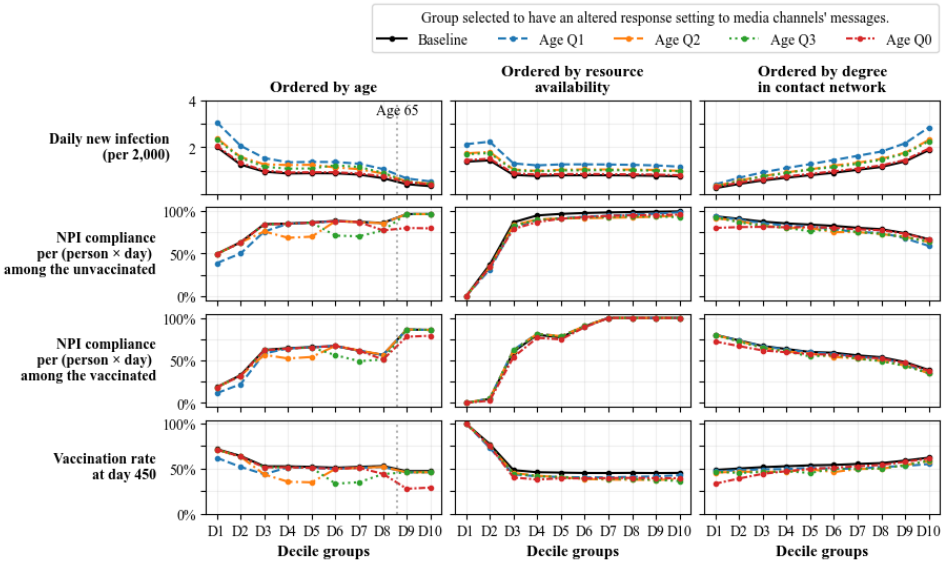

Fig C. Simulation results for different scenarios across each decile group in the subssection "Heterogeneous Responses to Intervention-Related Messages", where changes in response are imposed on groups characterized by age.

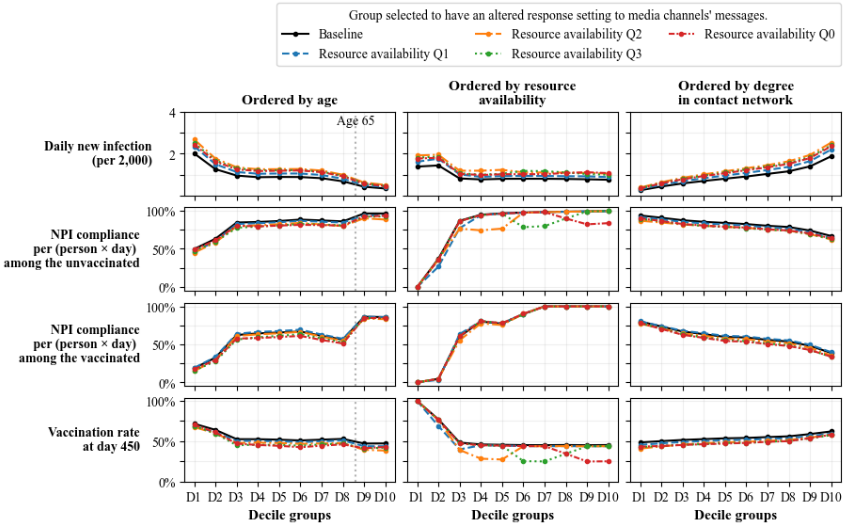

Fig D. Simulation results for different scenarios across each decile group in the subssection "Heterogeneous Responses to Intervention-Related Messages", where changes in response are imposed on groups characterized by resource availability.

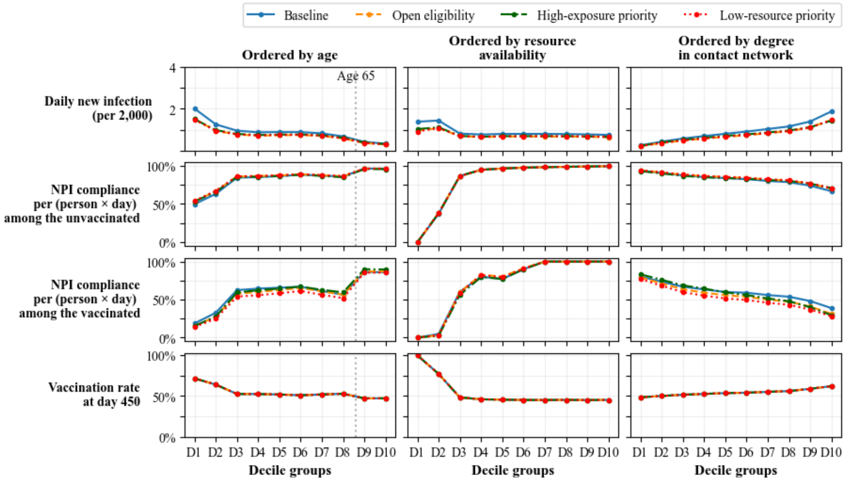

Fig E. Simulation results for different scenarios across each decile group in the subsection "Vaccine Eligibility Management."
