# Supplementary material for: Modeling individual self-protective behavior during epidemics
Source: PLoS Comput Biol. 2026 May 8;22(5):e1014252. doi: 10.1371/journal.pcbi.1014252 (PMC13170966; doi:10.1371/journal.pcbi.1014252)
Supplement: S3 Appendix — This appendix provides the parameter values used in the seven fitted models, organized into comprehensive tables. It includes parameter tuples that reproduce behavioral and epidemiological patterns observed in the calibration datasets. (PDF) [file pcbi.1014252.s003.pdf]

S3 Appendix. Calibration parameters: tables of definitions, ranges, and fitted values

Table A. Model parameters and their descriptions.

Symbol	Description
t_1	Timing of NPI mandate applied
t_2	Timing of NPI mandate lifted from the vaccinated
t_3	Timing of vaccine administration initiation
t_4	Timing of increase in vaccine administration capacity
$\mathcal{K}_1$	Vaccine administration capacity (phase 1)
$\mathcal{K}_2$	Vaccine administration capacity (phase 2)
$\alpha_k^{<65}$	Resource availability modifier for individuals aged 64 and younger when infected
α_k^{65+}	Resource availability modifier for individuals aged 65 and older when infected
$C^{(n)}$	NPI compliance cost (e.g., cost of supplies)
$C^{(p)}$	Non-compliance penalty
$C^{(q)}$	Discomfort cost due to congestion in the vaccine administration queue.
$\mu(F_k)$, $\sigma(F_k)$	Mean and standard deviation of the distribution of agents' pro-intervention media channel usage
$\mu(P_{t=0}^{(0,0)})$, $\sigma(P_{t=0}^{(0,0)})$	Mean and standard deviation of the distribution of agents' perceived probability of infection when not complying with NPIs and not vaccinated at $t = 0$; this perception evolves over time at the individual level as $P_{k,t}^{(0,0)}$.
$\mu(P_{t=0}^{(s)})$, $\sigma(P_{t=0}^{(s)})$	Mean and standard deviation of the distribution of agents' perceived probability of getting vaccine's side effect at $t = 0$; this perception evolves over time at the individual level as $P_{k,t}^{(s)}$.
$U_{\text{chan2}}^{(0,0)}$	Uncertainty value of channel 2 regarding its $P^{(0,0)}$ messages
$U_{\text{chan2}}^{(s)}$	Uncertainty value of channel 2 regarding its $P^{(s)}$ messages
$\mu_1^{(0,0)}$	Learning rate for $P_{k,t}^{(0,0)}$
$\mu_2^{(0,0)}$	Learning rate for $U_{k,t}^{(0,0)}$
$\mu_1^{(s)}$	Learning rate for $P_{k,t}^{(s)}$
$\mu_2^{(s)}$	Learning rate for $U_{k,t}^{(s)}$
$P^{(1,0)}$	Perceived probability of getting infected upon complying with NPI while not vaccinated (fixed).
$P^{(0,1)}$	Perceived probability of getting infected upon not complying with NPI while vaccinated (fixed).
$P^{(1,1)}$	Perceived probability of getting infected upon complying with NPI while vaccinated (fixed).
λ_0	Baseline transmission rate
ρ	Reduction rate provided by self-protection measures (e.g., face masks) upon NPI compliance
t_a	Start time of adjustments in the NPI's effectiveness (when one complied)
t_b	End time of adjustments in the NPI's effectiveness (when one complied)
δ	Reduction rate in the NPI's effectiveness during the time $[t_a, t_b]$
$\|I_0\|$	Initial number of infectious agents at the start of the simulation ($t = 0$).
σ	Probability that an exposed agent transitions to the infectious state ($E \rightarrow I$).
γ	Probability that an infectious agent recovers ($I \rightarrow R$).
ω	Probability that a recovered agent loses immunity and returns to the susceptible state ($R \rightarrow S$).

Table B. Parameter tuples used to reproduce patterns in the calibration datasets (Set 1)

Model	Fitted value															
	t_1	t_2	t_3	t_4	$\mathcal{K}_1$	$\mathcal{K}_2$	$\alpha_k^{<65}$	α_k^{65+}	$C^{(n)}$	$C^{(q)}$	$\mu(F_k)$	$\sigma(F_k)$	$\mu(P_{t=0}^{(0,0)})$	$\sigma(P_{t=0}^{(0,0)})$	$\mu(P_{t=0}^{(s)})$	$\sigma(P_{t=0}^{(s)})$
TB1 ($C^{(p)} = 100$)	102	421	255	343	85.0	103.0	0.9265	0.2351	126.9552	172.9456	0.82	0.04	0.04	0.01	0.39	0.16
TB2 ($C^{(p)} = 150$)	102	421	255	343	85.0	103.0	0.8239	0.3037	245.0718	224.5467	0.64	0.05	0.05	0.01	0.73	0.11
TB3 ($C^{(p)} = 200$)	102	421	255	343	85.0	103.0	0.7871	0.2238	322.2018	292.9102	0.61	0.07	0.04	0.01	0.47	0.17
TB4 ($C^{(p)} = 250$)	102	421	255	343	85.0	103.0	0.8475	0.2846	328.397	22.1407	0.62	0.09	0.04	0.01	0.45	0.16
TB5 ($C^{(p)} = 300$)	102	421	255	343	85.0	103.0	0.8365	0.3329	388.0384	2.531	0.66	0.09	0.04	0.01	0.45	0.14
TB6 ($C^{(p)} = 350$)	102	421	255	343	85.0	103.0	0.818	0.2833	475.798	110.0188	0.7	0.09	0.04	0.01	0.47	0.15
TB7 ($C^{(p)} = 400$)	102	421	255	343	85.0	103.0	0.7417	0.1247	546.6799	22.9374	0.82	0.04	0.05	0.01	0.47	0.17
Range	Fixed	Fixed	Fixed	Fixed	Fixed	Fixed	[0.5, 0.95]	[0.0, 0.50]	[0, 800]	[0, 400]	[0.60, 0.85]	[0.01, 0.12]	[0.02, 0.05]	Fixed	[0.25, 0.75]	[0.01, 0.18]

Table C. Parameter tuples used to reproduce patterns in the calibration datasets (Set 2)

Model	Fitted values								
	$U^{(0,0)}_{\text{channel 2}}$	$U^{(s)}_{\text{channel 2}}$	$\mu_1^{(0,0)}$	$\mu_2^{(0,0)}$	$\mu_1^{(s)}$	$\mu_2^{(s)}$	$P^{(1,0)}$	$P^{(0,1)}$	$P^{(1,1)}$
TB1 ($C^{(p)} = 100$)	0.143	0.2234	0.0382	0.1003	0.0729	0.2279	0.010	0.0555	0.0044
TB2 ($C^{(p)} = 150$)	0.2832	0.1507	0.0309	0.0552	0.1592	0.1664	0.010	0.0474	0.0036
TB3 ($C^{(p)} = 200$)	0.2453	0.1285	0.0559	0.1447	0.1406	0.2182	0.010	0.0513	0.0059
TB4 ($C^{(p)} = 250$)	0.2931	0.1436	0.0867	0.1987	0.1518	0.2500	0.010	0.0614	0.0062
TB5 ($C^{(p)} = 300$)	0.2977	0.1274	0.0949	0.2358	0.1431	0.2500	0.010	0.0682	0.0059
TB6 ($C^{(p)} = 350$)	0.298	0.1121	0.0947	0.1931	0.1365	0.2340	0.010	0.0845	0.0058
TB7 ($C^{(p)} = 400$)	0.2908	0.2995	0.0451	0.0924	0.0992	0.2397	0.010	0.0671	0.0050
Range	[0.1 , 0.3]	[0.1 , 0.3]	[0.01, 0.25]	[0.01, 0.25]	[0.01, 0.25]	[0.01, 0.25]	Fixed	[0.0, 0.10]	[0.0, 0.01]

Table D. Parameter tuples used to reproduce patterns in the calibration datasets (Set 3)

Basis	Fitted value								
	λ_0	ρ	t_a	t_b	δ	$\lvert I_0 \rvert$	σ	γ	ω
TB1 ($C^{(p)} = 100$)	0.0173	0.7225	189	268	0.2535	19	1/6.5	1/18	1/180
TB2 ($C^{(p)} = 150$)	0.0170	0.6996	212	263	0.3022	19	1/6.5	1/18	1/180
TB3 ($C^{(p)} = 200$)	0.0174	0.7221	189	268	0.2594	19	1/6.5	1/18	1/180
TB4 ($C^{(p)} = 250$)	0.0174	0.7264	189	268	0.2609	19	1/6.5	1/18	1/180
TB5 ($C^{(p)} = 300$)	0.0176	0.7212	189	267	0.2539	19	1/6.5	1/18	1/180
TB6 ($C^{(p)} = 350$)	0.0175	0.7171	189	267	0.2441	19	1/6.5	1/18	1/180
TB7 ($C^{(p)} = 400$)	0.0173	0.7062	214	264	0.2976	19	1/6.5	1/18	1/180
Range	[0.016, 0.018]	[0.60, 0.99]	[180, 220]	[220, 320]	[0.20, 0.35]	Fixed	Fixed	Fixed	Fixed
Remark	[1]	[2]	-	-	-	[3]	[4]	[5]	[6]

References

1. Vazquez A, Staebler M, Khanin A, Lichte D, Brucherseifer E. Estimating the super-spreading rate at workplaces using bluetooth technology. medRxiv. 2021; p. 2021–03.

2. Wang Y, Deng Z, Shi D. How effective is a mask in preventing COVID-19 infection? Medical devices & sensors. 2021;4(1):e10163.

3. Yu G, Garee M, Ventresca M, Yih Y. How individuals' opinions influence society's resistance to epidemics: an agent-based model approach. BMC Public Health. 2024;24(1):863.

4. Alene M, Yismaw L, Assemie MA, Ketema DB, Gietaneh W, Birhan TY. Serial interval and incubation period of COVID-19: a systematic review and meta-analysis. BMC infectious diseases. 2021;21:1–9.

5. Byrne AW, McEvoy D, Collins AB, Hunt K, Casey M, Barber A, et al. Inferred duration of infectious period of SARS-CoV-2: rapid scoping review and analysis of available evidence for asymptomatic and symptomatic COVID-19 cases. BMJ open. 2020;10(8):e039856.

6. Dan JM, Mateus J, Kato Y, Hastie KM, Yu ED, Faliti CE, et al. Immunological memory to SARS-CoV-2 assessed for up to 8 months after infection. Science. 2021;371(6529):eabf4063.
