# Supplementary material for: Modeling individual self-protective behavior during epidemics
Source: PLoS Comput Biol. 2026 May 8;22(5):e1014252. doi: 10.1371/journal.pcbi.1014252 (PMC13170966; doi:10.1371/journal.pcbi.1014252)
Supplement: S4 Appendix — The table of dates on which various U.S. states expanded COVID-19 vaccine eligibility to specific age groups. The threshold ages (lower bounds) are specified in the table header. This information reflects the phased approach states adopted to distribute vaccines during the pandemic, prioritizing older populations before gradually including younger age groups. (PDF) [file pcbi.1014252.s004.pdf]

S4 Appendix. COVID-19 vaccine eligibility timelines by age group and U.S. state.

Table A. The table of dates on which various U.S. states expanded COVID-19 vaccine eligibility to specific age groups. The threshold ages (lower bounds) are specified in the table header. This information reflects the phased approach states adopted to distribute vaccines during the pandemic, prioritizing older populations before gradually including younger age groups [1]

State	16+	30+	40+	45+	50+	55+	60+	65+	70+	75+	80+
Alabama	4/5/2021					3/22/2021		2/8/2021		1/8/2021	
Alaska	3/9/2021					3/3/2021					
Arizona	3/24/2021					3/4/2021		1/19/2021		12/28/2020	
Arkansas	3/30/2021							3/8/2021	1/18/2021	1/5/2021	
California	4/15/2021				4/1/2021			1/13/2021			
Colorado	4/2/2021				3/29/2021		3/5/2021	2/8/2021	1/29/2021		
Connecticut	4/1/2021			3/19/2021		3/1/2021		2/11/2021		1/18/2021	
Delaware	4/6/2021				3/17/2021			1/19/2021			
Florida	4/5/2021		3/29/2021		3/22/2021		3/15/2021	1/19/2021			
Georgia	3/25/2021					3/15/2021		12/30/2020			
Hawaii	4/19/2021				4/12/2021		3/29/2021	3/15/2021	3/8/2021	2/19/2021	
Idaho	4/5/2021							2/1/2021			
Illinois	4/12/2021							1/25/2021			
Indiana	3/31/2021	3/29/2021	3/22/2021		3/3/2021	3/2/2021	2/23/2021	2/1/2021	1/13/2021		
Iowa	4/5/2021							2/1/2021			
Kansas	3/29/2021							1/21/2021			
Kentucky	4/5/2021						3/1/2021		2/1/2021		
Louisiana	3/29/2021							2/8/2021	1/4/2021		
Maine	4/7/2021				3/23/2021		3/3/2021				
Maryland	4/6/2021						3/23/2021			1/18/2021	
Massachusetts	4/19/2021						3/22/2021			2/1/2021	
Michigan	4/5/2021				3/22/2021						
Minnesota	3/30/2021										
Mississippi	3/16/2021				3/4/2021					1/6/2021	
Missouri	4/9/2021							1/18/2021			
Montana	4/1/2021						3/8/2021		1/19/2021		
Nebraska	4/5/2021				3/22/2021						
Nevada	4/5/2021								1/11/2021		
New Hampshire	4/2/2021	3/31/2021	3/29/2021					1/26/2021			
New Jersey	4/19/2021					4/5/2021		1/14/2021			
New Mexico	4/5/2021										
New York	4/6/2021	3/30/2021			3/23/2021		3/10/2021	1/12/2021		1/11/2021	
North Carolina	4/7/2021									12/30/2020	
North Dakota	3/29/2021										
Ohio	3/29/2021		3/19/2021		3/11/2021		3/4/2021	2/8/2021	2/1/2021	1/25/2021	1/19/2021
Oklahoma	3/29/2021										
Oregon	4/19/2021								2/22/2021		2/8/2021
Pennsylvania	4/13/2021							1/19/2021		1/8/2021	
Rhode Island	4/19/2021				4/5/2021			2/22/2021		1/28/2021	
South Carolina	3/31/2021					3/8/2021		2/8/2021	1/13/2021		
South Dakota	4/5/2021									2/8/2021	
Tennessee	4/5/2021										
Texas	3/29/2021				3/15/2021			2/18/2021	1/11/2021		
Utah	3/24/2021				3/4/2021						
Vermont	4/19/2021	4/12/2021	4/5/2021		3/29/2021		3/25/2021	3/1/2021	2/16/2021	1/25/2021	
Virginia	4/18/2021									1/11/2021	
Washington	4/15/2021							1/18/2021			
West Virginia	3/22/2021				3/3/2021			1/19/2021	1/13/2021		
Wisconsin	4/5/2021							1/25/2021			
Wyoming	3/31/2021										

References

1. Ballotpedia. Coronavirus (COVID-19) Vaccination Rates and Distribution Plans by State; 2020. Available from: `https://ballotpedia.org/Coronavirus_(COVID-19)_vaccination_rates_and_distribution_plans_by_state`.
