# Supplementary material for: Modeling individual self-protective behavior during epidemics
Source: PLoS Comput Biol. 2026 May 8;22(5):e1014252. doi: 10.1371/journal.pcbi.1014252 (PMC13170966; doi:10.1371/journal.pcbi.1014252)
Supplement: S5 Appendix — This supporting information provides additional results from the variance-based global sensitivity analysis conducted using Sobol indices which quantifies how uncertainty in model parameters contributes to variability in model outcomes. (PDF) [file pcbi.1014252.s005.pdf]

S5 Appendix. Variance-based global sensitivity analysis (Sobol indices)

This appendix provides additional methodological details and results from the variance-based global sensitivity analysis conducted using Sobol indices [1]. The analysis is conducted to quantify how uncertainty in model parameters contributes to variability in model outcomes. This approach decomposes the total variance of a model output into contributions attributable to individual parameters and their interactions.

For each parameter, we computed the first-order Sobol index ($S1$), which measures the proportion of output variance explained by variation in that parameter alone, holding all other parameters fixed on average. In contrast, the total-effect Sobol index (ST) measures the proportion of output variance attributable to a parameter including all interaction effects with other parameters. As such, ST captures both the direct (main) effect of a parameter and its contributions through higher-order interactions.

We used the Python package `SALib` (version 1.5.2), which employs Sobol sampling of the parameter space based on a quasi-random low-discrepancy sequence. Parameter sets were generated using a base sample size of $N = 512$. Because second-order Sobol indices were not evaluated in this appendix, the total number of model evaluations (particles) was 15,360, following the standard first-order Sobol sampling design.

Sensitivity analyses were conducted for nine outcome measures: (i) the total number of new infections for the overall population, as well as for the oldest 20% age group and the lowest 20% resource-availability group; (ii) the NPI compliance rate for the same three populations; and (iii) the final vaccination coverage for the same three populations. These subgroup-specific analyses were motivated by the primary focus of the main manuscript on outcome heterogeneity among older individuals and populations with limited resource availability. Accordingly, this supporting document examines how uncertainty in model parameters propagates to variation in key epidemiological and behavioral outcomes within these priority groups. To reduce computational burden, sensitivity analysis was performed using a single calibrated model configuration rather than all seven fitted models. Specifically, we selected the configuration with $C^{(P)} = 250$ (non-compliance penalty), which provided the best overall fit during model calibration.

Model parameters and bounds used in the Sobol global sensitivity analysis. Most of the parameter ranges are from the model calibration range that is specified in Appendix C. You can also find the descriptions of parameters in the same document. We added t_1, t_2, t_3, and t_4 which determines the timings of events in our simulation model and $\mathcal{K}_1$ and $\mathcal{K}_2$ which determines the vaccine administration capacities to the sensitivity analysis. These were fixed in the calibration setting but we allowed them to have 2 month ranges for timing parameters and $\pm 20\%$ ranges for capacity parameters. These newly added ranges are listed in Table A.

Table A. Model parameters and bounds used in the Sobol global sensitivity analysis that were not included in the calibration ranges reported in Appendix C.

Symbol	Lower	Upper	Description
t_1	72	132	Timing of NPI mandate applied
t_2	391	451	Timing of NPI mandate lifted from the vaccinated
t_3	225	285	Timing of vaccine administration initiation
t_4	313	373	Timing of increase in vaccine administration capacity
K_1	68	102	Vaccine administration capacity (phase 1)
K_2	81.5	124.5	Vaccine administration capacity (phase 2)

1 Global sensitivity analysis for total new infections

The first part of the analysis examines first-order ($S1$) and total-effect (ST) Sobol indices for total new infections, evaluated for the overall population, the oldest 20% age group, and the lowest 20% resource-availability group. Fig A presents the results, showing the ten parameters with the largest total-effect values.

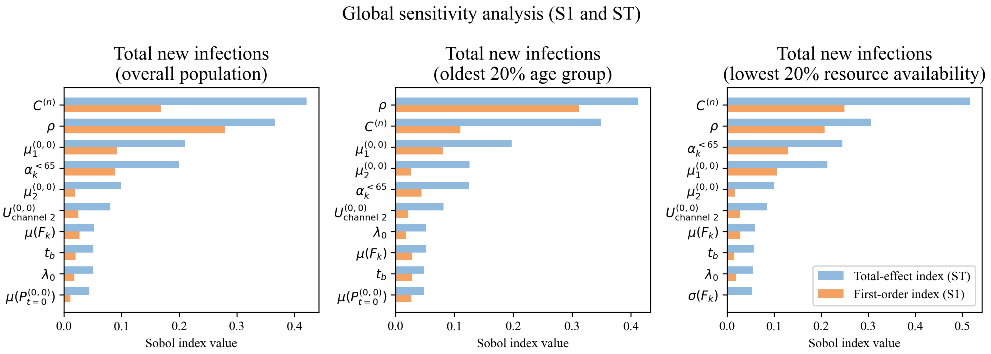

Fig A. Global sensitivity analysis for total new infections. First-order and total-effect Sobol indices quantify parameter contributions to variance in total new infections, evaluated for the overall population, the oldest 20% age group, and the lowest 20% resource-availability group.

Across all three populations, $C^{(n)}$ (NPI compliance cost) exhibits the largest total-effect index, indicating that uncertainty in the perceived cost of complying with NPIs is the dominant driver of variability in cumulative infections. Given that agents' decision-making is based on a cost–benefit perspective implemented through decision trees, this result is expected. The pronounced gap between ST and $S1$ suggests that the effect of $C^{(n)}$ is largely interaction-driven, operating jointly with other parameters rather than through a purely additive effect. This reflects the presence of multiple components in the decision tree and other parts of the model that can interact with $C^{(n)}$.

ρ (reduction rate provided by self-protection measures upon NPI compliance) consistently ranks among the most influential parameters across all panels. Its substantial first-order contribution indicates a strong direct effect on infection outcomes, consistent with its role in modulating how infection risk accumulates over time during the exposure process.

Finally, learning rates associated with agents' perceived infection risk ($P^{(0,0)_{k,t}}$) and the resource availability modifiers ($\alpha_k^{<65}$) contribute moderately to infection variability. The influence of $\alpha_k^{<65}$ is more pronounced in the lowest 20% resource-availability group, reflecting heightened sensitivity of infection outcomes in resource-limited settings.

2 Global sensitivity analysis for NPI compliance rate

Fig B presents Sobol indices for the NPI compliance rate, evaluated for the overall population, the oldest 20% age group, and the lowest 20% resource-availability group. Overall, the sensitivity patterns for compliance are broadly consistent with those observed for total new infections, with cost-related parameters playing a dominant role and interaction effects varying across subpopulations.

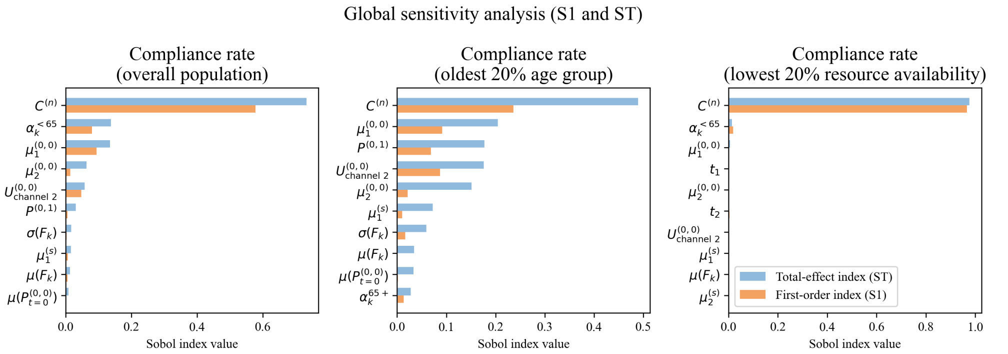

Fig B. Global sensitivity analysis for NPI compliance rate. First-order and total-effect Sobol indices quantify parameter contributions to variance in NPI compliance rate, evaluated for the overall population, the oldest 20% age group, and the lowest 20% resource-availability group.

The third panel highlights the dominant role of $C^{(n)}$ among the lowest 20% resource-availability group, indicating that uncertainty in the cost of NPI compliance overwhelmingly explains variation in compliance behavior within this subgroup. This result suggests that, for agents with limited resources, NPI decision-making is driven primarily by cost considerations, with other behavioral, informational, or learning-related factors playing a negligible role. In contrast, the oldest 20% age group exhibits a more distributed sensitivity profile, with multiple parameters contributing to outcome variance through interaction effects. Although $C^{(n)}$ remains an important driver of compliance, it no longer fully dominates the variance, indicating that compliance behavior in this subgroup is shaped by a broader set of factors, including learning dynamics and perceived infection risk.

As expected, the transmission-related parameter ρ does not appear among the top ten parameters when the target outcome is compliance rate. In the model, ρ influences disease transmission dynamics but does not directly affect agents' NPI compliance decisions, which are rather governed by cost–benefit considerations, perceived risks, and opinion learning processes.

3 Global sensitivity analysis for final vaccination rate

Fig C presents Sobol indices for final vaccination coverage or the vaccination rate at the end of each simulation run, evaluated for the overall population, the oldest 20% age group, and the lowest 20% resource-availability group. For both the overall population and the oldest 20% age group, the two learning rates that govern how agents update their perceived probability of vaccine side effects dominate variability in final vaccination coverage. Its large first-order contribution indicates that uncertainty in how quickly agents adapt their vaccine-related risk perceptions has a strong direct effect on vaccination uptake. This pattern highlights the central role of opinion dynamics and learning processes in shaping vaccination behavior, particularly among older individuals.

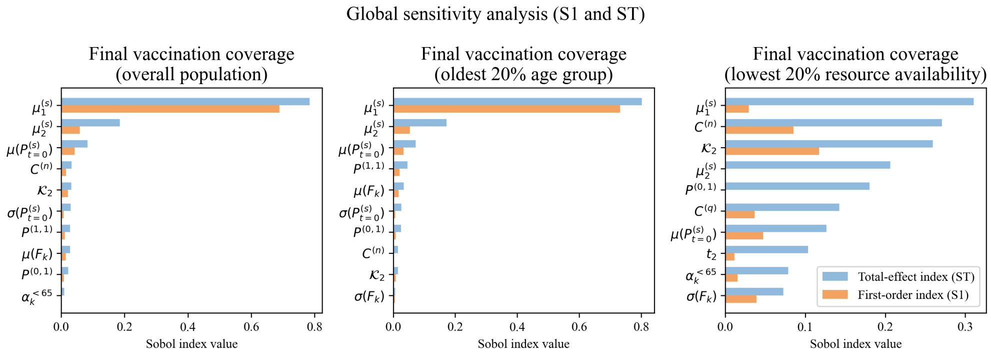

Fig C. Global sensitivity analysis for final vaccination rate. First-order and total-effect Sobol indices quantify parameter contributions to variance in the final vaccination rate at the end of the simulation, evaluated for the overall population, the oldest 20% age group, and the lowest 20% resource-availability group.

Secondary contributors in these two panels include the mean of the initial opinion distribution of $P_{k,t}^{(s)}$, suggesting that the initial distribution of vaccine-risk perceptions influence eventual coverage. In contrast, cost-related parameters such as $C^{(n)}$ play a comparatively minor role in determining vaccination outcomes for these populations.

A markedly different sensitivity structure emerges for the lowest 20% resource-availability group. In this subgroup, vaccination coverage is influenced by a broader set of parameters, with $C^{(n)}$ (NPI compliance cost) and $\mathcal{K}_2$ (vaccine administration capacity during the expanded rollout phase) exhibiting substantial total-effect indices. This indicates that, among resource-constrained populations, vaccination uptake is shaped not only by learning and perception dynamics but also by cost-related and capacity-related constraints.

Overall, the sensitivity patterns for vaccination coverage differ from those observed for total infections and compliance rates. While cost-related parameters dominate compliance and infection outcomes, learning dynamics related to vaccine risk perception dominate vaccination uptake in the general and older populations. In contrast, vaccination behavior in resource-limited populations reflects a combination of behavioral learning, cost constraints, and logistical capacity, underscoring the importance of addressing both informational and structural barriers to improve vaccine coverage.

References

1. Sobol IM. Global sensitivity indices for nonlinear mathematical models and their Monte Carlo estimates. Mathematics and computers in simulation. 2001;55(1-3):271–280.
