# Supplementary material for: Modeling individual self-protective behavior during epidemics
Source: PLoS Comput Biol. 2026 May 8;22(5):e1014252. doi: 10.1371/journal.pcbi.1014252 (PMC13170966; doi:10.1371/journal.pcbi.1014252)
Supplement: S6 Appendix — This supporting information provides additional results for a scenario without media influence, NPI mandates, and vaccine introduction, designed to explore the scenario where opinion dynamics driven solely by peer interactions. (PDF) [file pcbi.1014252.s006.pdf]

S6 Appendix. Supplementary scenario without media influence, NPI mandates, or vaccine introduction

This supporting information provides additional results for a scenario without media influence, NPI mandates, and vaccine introduction. In this setting, no entities actively intervene to influence public opinion or shape consensus regarding the disease, NPIs, or vaccine side effects. Instead, agents form and update their opinions solely through peer interactions within their social networks.

This scenario represents a stricter interpretation of "no intervention" than those considered in the main analysis. It is included as an additional exploratory scenario to isolate peer-driven opinion dynamics, rather than as a core scenario aligned with the primary research questions. In real-world settings, information dissemination through media channels and influential actors is pervasive and difficult to eliminate entirely, making this scenario primarily illustrative.

In this setting, behavioral responses emerge solely from peer-driven opinion dynamics, without reinforcement from external interventions or media influence. As shown in Fig A, NPI compliance evolves more gradually and remains lower compared to observed data, which is reproduced by scenarios with mandates or media influence in the main text, while vaccination coverage remains zero by design (no vaccine). This pattern highlights the role of media channels as opinion leaders in our model. The resulting disease dynamics reflect the absence of coordinated behavioral responses, highlighting the role of external signals in accelerating and amplifying collective action.

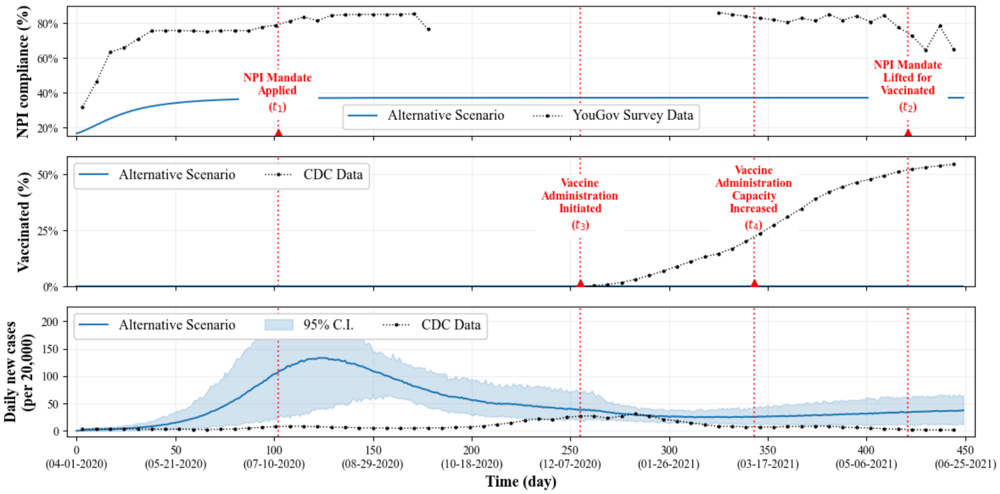

Fig A. Simulation results for the supplementary scenario without media influence, NPI mandates, or vaccine introduction. Trends in NPI compliance (top), vaccination coverage (middle), and daily new cases (bottom) are shown for a setting in which agents rely solely on peer interactions to update opinions.
